# How pre-processing decisions affect the reliability and validity of the approach–avoidance task: Evidence from simulations and multiverse analyses with six datasets

**DOI:** 10.3758/s13428-023-02109-1

**Published:** 2023-05-23

**Authors:** Sercan Kahveci, Mike Rinck, Hannah van Alebeek, Jens Blechert

**Affiliations:** 1 Department of Psychology, Paris-Lodron-University of Salzburg, Hellbrunner Straße 34, 5020 Salzburg, Austria; 2 Centre for Cognitive Neuroscience, Paris-Lodron-University of Salzburg, Salzburg, Austria; 3 Behavioural Science Institute, Radboud University, Nijmegen, The Netherlands

**Keywords:** Approach-avoidance task (AAT), Bias scores, Reliability, Validity, Outlier exclusion, Simulation, Multiverse analysis

## Abstract

**Abstract:**

Reaction time (RT) data are often pre-processed before analysis by rejecting outliers and errors and aggregating the data. In stimulus–response compatibility paradigms such as the approach–avoidance task (AAT), researchers often decide how to pre-process the data without an empirical basis, leading to the use of methods that may harm data quality. To provide this empirical basis, we investigated how different pre-processing methods affect the reliability and validity of the AAT. Our literature review revealed 108 unique pre-processing pipelines among 163 examined studies. Using empirical datasets, we found that validity and reliability were negatively affected by retaining error trials, by replacing error RTs with the mean RT plus a penalty, and by retaining outliers. In the relevant-feature AAT, bias scores were more reliable and valid if computed with D-scores; medians were less reliable and more unpredictable, while means were also less valid. Simulations revealed bias scores were likely to be less accurate if computed by contrasting a single aggregate of all compatible conditions with that of all incompatible conditions, rather than by contrasting separate averages per condition. We also found that multilevel model random effects were less reliable, valid, and stable, arguing against their use as bias scores. We call upon the field to drop these suboptimal practices to improve the psychometric properties of the AAT. We also call for similar investigations in related RT-based bias measures such as the implicit association task, as their commonly accepted pre-processing practices involve many of the aforementioned discouraged methods.

**Highlights:**

• Rejecting RTs deviating more than 2 or 3 SD from the mean gives more reliable and valid results than other outlier rejection methods in empirical data

• Removing error trials gives more reliable and valid results than retaining them or replacing them with the block mean and an added penalty

• Double-difference scores are more reliable than compatibility scores under most circumstances

• More reliable and valid results are obtained both in simulated and real data by using double-difference D-scores, which are obtained by dividing a participant’s double mean difference score by the SD of their RTs

## Introduction

Stimulus–response compatibility tasks like the approach–avoidance task (AAT; Solarz, 1960), the extrinsic affective Simon task (De Houwer, 2003), and the implicit association task (IAT; Greenwald et al., 1998) have been used for over 60 years to measure attitudes without directly asking the participant. Their strength lies in the fact that they measure stimulus–response compatibility implicitly through reaction times (RTs), which avoids the methodological issues associated with self-reports, such as social desirability and experimenter demand. In turn, however, they are subject to all the methodological issues associated with RT tasks, such as occasional incorrect responses, outlying RTs, and the large quantity of data, which cannot be meaningfully interpreted until it is reduced. As such, the data usually undergo some kind of pre-processing before analysis, whereby error trials and outliers are dealt with in some manner (or not), often followed by aggregation of the data into an easily interpretable bias score.

There are many methods available to perform each of these pre-processing steps. However, there is no clear-cut answer on which methods are preferable and under which circumstances, leaving researchers to find their way through this garden of forking paths on their own. Decisions may be made on the basis of their effect on the data, thereby inflating the likelihood of obtaining spurious results (Gelman & Loken, 2013). Researchers may choose the same pre-processing pipeline as in already published work. This allows for comparable results, but makes the quality of the findings of an entire line of research dependent on the efficiency of the most popular pre-processing pipeline. In the best case, the commonly accepted set of decisions reliably recover the true effect and allow the field to progress based on grounded conclusions. In the worst case, it makes the measurement less reliable, thereby misleading researchers with conclusions based on random noise and null findings that mask true effects. Hence, both heterogeneity in pre-processing decisions as well as low reliability can contribute to inconsistent results across studies that investigate the exact same effect, and thus play a role in the ongoing replication crisis in psychological science. Ideally, pre-processing decisions would be made based on empirical findings that demonstrate which options yield the best results (see e.g. Berger & Kiefer, 2021; Ratcliff, 1993).

The literature on the AAT is no stranger to these issues. The field did not take up the methods which Krieglmeyer and Deutsch (2010) found to lead to the highest reliability and validity (i.e. either strict slow RT cutoffs or data transformation). Many labs have since settled into their own pre-processing pipeline without a firm empirical basis for their decisions, making it unclear whether differing results are due to differences in task setup or in pre-processing after data collection. For example, using the same task setup in which participants approach and avoid stimuli with a joystick, one study found a correlation between spider fear and spider avoidance bias (e.g. Reinecke et al., 2010), while another did not (e.g. Krieglmeyer & Deutsch, 2010). It is unclear whether this difference occurred because the former study did not remove outliers whereas the latter removed all RTs above 1500 ms, or because the former study featured 2.66 times more test trials and 2.25 times more practice trials than the latter, or because the two studies used different scoring algorithms.

Low reliability has also been a problem in the AAT literature (Loijen et al., 2020), at least for certain variants of it. The irrelevant-feature AAT manipulates the contingency between the approach or avoidance response and a task-irrelevant stimulus feature, for example, by measuring chocolate approach–avoidance bias by requiring participants to approach stimuli surrounded by a green frame and avoid stimuli surrounded by a blue frame, thereby making it irrelevant whether the stimulus itself contains chocolate. This task is reported in the literature as unreliable, with reliabilities below zero (Kahveci, Van Bockstaele, et al., 2020; Lobbestael et al., 2016; Wittekind et al., 2019), though reliabilities around .40 (Cousijn et al., 2014) and even .80 have been reported on individual occasions (Machulska et al., 2022). It has seen frequent use, because its indirect nature conceals the goal of the experiment and thus makes it less susceptible to experimenter demand. The relevant-feature AAT, in contrast, directly manipulates the contingency between a task-relevant feature of the stimulus and the response, for example, by measuring chocolate approach–avoidance bias by requiring participants to approach chocolate stimuli during one block and to avoid it during another block. This task usually has a high reliability, from around .90 (Zech et al., 2022), to around .70 (Hofmann et al., 2009; Van Alebeek et al., 2021), up to .50 (Kahveci, Van Bockstaele, et al., 2020); however, the direct nature of its instructions make it easy for the participant to figure out what the task is about.

In the present study, we probed the extent of pre-processing heterogeneity in the literature on the AAT, and we made an effort towards reducing it by examining the reliability and validity obtained through a wide range of pre-processing decisions using a *multiverse analysis*, thereby limiting the range of acceptable pre-processing methods to only the most reliable and valid approaches. The multiverse analysis methodology, advocated by Steegen et al. (2016), involves applying every combination of reasonable analysis decisions to the data, to probe how variable the analysis outcomes can be, and to what extent each analysis decision contributes to this variability. We know of one study so far that has examined the impact of pre-processing methods on the reliability of the AAT, though it did not utilize multiverse analysis. Krieglmeyer and Deutsch (2010) applied a number of different methods to deal with outliers to the data and compared the resulting bias scores on the basis of their split-half reliability and overall effect size, finding that the relevant-feature AAT is most reliable when no outlier correction is applied, while the irrelevant-feature AAT benefits from very strict outlier rejection, e.g. removing all RTs above 1000 ms or deviating more than 1.5 *SD*s from the mean. Additionally, Parsons (2022) was the first to examine the effect of pre-processing decisions on reliability, though he looked at the dot-probe, Stroop, and Flanker tasks rather than the AAT.

Our study instead focused on the AAT, but also extended these studies methodologically by also examining criterion validity, as high reliability is a prerequisite, but not a guarantee for high validity; if we focused solely on reliability, we would risk achieving highly reliable, but invalid data. Reliability represents how well a measurement will correlate with the same measurement performed again (Spearman, 1904), but it is agnostic on what is actually measured. Hence, one could measure something reliably, but that something might be an artifact rather than the effect one was looking for. For example, participants tend to be slower in the beginning of the experiment when they are trying to adapt to the task, and some are slower than others. This initial slowness is a large interpersonal difference that can be measured reliably, but it has little to do with cognitive bias. If we only focus on reliability, we may erroneously believe that our analysis should focus on this initial slowness rather than ignore it.

We also extended these previous studies by examining simulated as well as real data: simulated data allows for a detailed analysis of the conditions under which different outlier rejection and bias scoring methods are more or less reliable; but only real data can be used to examine how validity is affected by bias scoring and error and outlier handling. Previous simulation studies examining outlier rejection have assumed that extreme RTs are unrelated to the individual’s actual underlying score (Berger & Kiefer, 2021); if, in real data, the approach–avoidance bias expresses itself through errors and extreme RTs (e.g. stronger bias leading to more errors when avoiding desired stimuli), then it could turn out to be preferable to keep them in the data.

### Data structure and methodological challenges of the AAT

In this section, we will discuss the characteristics of the AAT to understand the methodological challenges that will need to be addressed when pre-processing its data. In the AAT, participants view different stimuli and give either a speeded approach or avoidance response depending on a feature of the stimulus. Responses are typically given with a joystick or a similar device that simulates approach toward or avoidance of a given stimulus (Wittekind et al., 2021), though simple buttons are sometimes used instead. Depending on the input device, this allows for the measurement of different types of response times per trial, which we term initiation time, movement duration, and completion time (terms previously used by: Barton et al., 2021; Tzavella et al., 2021). The time from stimulus onset to shortly after response onset (initiation time) indicates how long it took the participant to initiate a response; the time from response onset until response completion (movement duration) indicates the speed of the approach or avoidance movement. The two are often added together to represent the latency from stimulus onset until response completion (completion time). Approach–avoidance bias scores quantify the extent to which responses slow down or speed up due to the compatibility between stimulus and response.

A typical AAT trial features one out of two stimulus categories (target and control) and requires a response in one out of two directions (approach and avoid), resulting in four different types of trials. RTs to these four types of trials can be decomposed into three independent contrasts, which are detailed in Table 1. The first contrast is the RT difference between responses to the two stimulus categories (rows in Table 1), regardless of response direction. This difference can be caused by the familiarity, visual characteristics, or the prototypicality of the stimulus as a stand-in for its stimulus category, among other causes. As shown in Table 1, this factor contaminates any difference score between single-direction responses to one stimulus category versus another. If this is ignored, we may erroneously conclude that a familiar stimulus category is approached faster than a less familiar category, even though all responses to the familiar stimulus category are faster, regardless of response direction.

**Table 1:** AAT trial types, difference scores, and their components

		Movement direction				
		Avoid		Approach		
Stimulus category	Target	(Quadrant A) Target stimulus recognition + general avoid speed + avoidance facilitation of target	–	(Quadrant B) Target stimulus recognition + general approach speed + approach facilitation of target	=	*Target-specific difference score* = (General avoid speed –general approach speed) + (avoidance facilitation of target – approach facilitation of target)
		–		–		–
	Control	(Quadrant C) Control stimulus recognition + general avoid speed + avoidance facilitation of control	–	(Quadrant D) Control stimulus recognition + general approach speed + approach facilitation of control	=	*Control-specific difference score* = (General avoid speed – general approach speed) + (avoidance facilitation of control – approach facilitation of control)
		=		=		=
		*(negative) Avoid-specific difference score* = (target stimulus recognition – control stimulus recognition) + (avoidance facilitation of target – avoidance facilitation of control)	–	*(negative) Approach-specific difference score* = (target stimulus recognition – control stimulus recognition) + (approach facilitation of target – approach facilitation of control)	=	*Double-difference score* = (avoidance facilitation of target – approach facilitation of target) – (avoidance facilitation of control – approach facilitation of control)

*Note:* This table is a schematic depiction of single- and double-difference scores and the RT components they consist of. The quadrants contain a description of which RT components we hypothesize to constitute the RTs of the combination of stimulus and response that the quadrant represents. When read from top to bottom, the bottom row represents the result of subtracting the middle row from the top row. When read from left to right, the right column represents the result of subtracting the middle column from the left column

The second contrast is the RT difference between approach and avoidance trials, regardless of stimulus content (columns in Table 1). This difference can be caused by the relative ease with which approach and avoidance movements can be made, which can be influenced by irrelevant factors like the individual’s anatomy and posture as well as by the (biomechanical) setup of the response device. This factor contaminates any difference score between approach and avoid trials within a single stimulus category. For example, a study found that patients with anorexia nervosa avoid, rather than approach, thin female bodies (Leins et al., 2018). Does that mean that women with anorexia, counter-intuitively, have an avoidance bias away from these stimuli? Such an interpretation would not be valid, since an identical avoidance bias was demonstrated for normal-weight bodies in the same patient group as well as in healthy individuals, indicating that avoidance responses were simply faster and not specific to thin bodies.

The third contrast is the approach–avoidance bias, and is represented by the difference between approaching and avoiding a target stimulus category, relative to the difference between approaching and avoiding a reference stimulus category. As shown in Table 1, this *double difference* can be interpreted as an approach or avoidance bias towards one particular stimulus type relative to another.

### The current study

The current article consists of four studies. As a first step, we reviewed the literature to gain insight into which pre-processing decisions are in use in the field (*Study 1*). We discuss thereafter which methods are potentially problematic and consider alternative methods, giving extra consideration to robust and novel approaches. Next, we performed a simulation study to compare two ways of aggregating data from four conditions, those being double-difference scores and compatibility scores (*Study 2*). We followed up with a simulation study to compare the impact of outliers on the reliability of scores derived using a number of outlier detection methods and scoring algorithms (*Study 3*). And lastly, we compared these pre-processing methods on how they affect the reliability and validity of real datasets in a multiverse analysis (*Study 4*).

## Study 1: Literature review

### Introduction

We performed a focused scoping review of the AAT literature to examine which pre-processing decisions are used in the field of AAT research. The intention was not to be exhaustive or systematic but to tap into the variability in pre-processing decisions in the field to orient the rest of this project.

### Methods

We reviewed 213 articles retrieved from Google Scholar using the keywords “approach–avoidance task OR approach avoidance task” published between 2005 and 2020. We rejected 65 articles after reading the abstract or full text, since they featured no AAT, only a training-AAT, or a variant of the AAT that departs strongly from the original paradigm (e.g. by allowing participants to freely choose whether to approach or avoid a stimulus). We also excluded one experiment which featured multiple pre-processing tracks, as we would otherwise have to count two full pre-processing pipelines for a single experiment. We thus retained 143 articles containing a total of 163 AATs. When an article contained multiple AAT experiments, all were included as separate entries and counted as such. The experiments were coded on the following variables: instruction type (relevant-feature, irrelevant-feature), response device (e.g. joystick, keyboard, mouse), RT definition (initiation time, movement duration, completion time), inclusion of some sort of training or therapy, the research population, the target stimuli, the type of reported reliability index if any (e.g. even-odd split-half, Cronbach’s alpha of stimulus-specific bias scores), absolute outlier exclusion rules (e.g. any RTs above 2000 ms), adaptive outlier exclusion rules (e.g. 3 SD above each participant’s mean), error handling rules (e.g. include error trials in analyses, force participants to give correct responses), performance-based participant exclusion rules (e.g. more than 35% errors), score-based exclusion rules (e.g. bias scores deviating more than 3 SD from sample mean), and the summary statistic used (e.g. double mean difference scores, median category-specific difference scores, simple means).

### Results

A total of 163 experiments from 143 articles were examined. Below, we describe the number and percentage of experiments that utilized specific methods in their design, pre-processing, and analysis. The full results of this review can be found in this study’s online repository: 10.17605/OSF.IO/YFX2C

#### Response device

Joysticks were by far the most popular response device (132; 80%). They were followed by keyboards (8; 4.91%), button boxes (7; 4.29%), touchscreens (5; 3.07%), computer mice (5; 3.07%), and other/multiple/unknown devices (8; 4.91%).

#### Instructions

The irrelevant-feature AAT was the most popular task type (119; 73.01%), followed by the relevant-feature AAT (41; 25.15%). A small number (3; 1.84%) used both task types in the same experiment.

#### Reliability measures

Reliability was not examined in the majority of experiments (125; 76.69%); most that did examine reliability used a single reliability measure (36; 22.1%), and some used two (2; 1.23%). Split-half reliability was the most common measure (19; 11.7%). The types of split-half reliability included temporal split-half, which is splitting the experiment halfway (5; 3.07%); even-odd split-half, which is splitting the data by even-uneven trial number (5; 3.07%); and randomized split-half, which is averaging together the correlations between many random splits (5; 3.07%); other studies did not mention the type of split-half used (4; 2.45%). Cronbach’s alpha was the next most common reliability measure (16; 9.82%). Most experiments computed Cronbach’s alpha on the basis of the covariance matrix of stimulus-specific bias scores (11; 6.75%), while a minority computed Cronbach’s alpha for RTs in a single movement direction, grouping them per stimulus (2; 1.23%), and some did not clarify how they computed Cronbach’s alpha (3; 1.84%). The least common measure was test-retest reliability (4; 2.45%).

#### RT measures

Most studies used a single RT measure (153; 93.9%) but some used multiple (10; 6.13%). Out of all examined experiments, most did not report how RTs were defined (69; 42.33%), but those that did used completion time (50; 30.7%), initiation time (43; 26.4%), or movement duration (9; 5.52%).

#### Outlier rejection rules

Many experiments applied no outlier rejection (62; 38%), while those that did either applied only absolute outlier rejection methods (38; 23.3%), only adaptive outlier rejection methods (24; 14.7%), or both together (39; 23.9%). Frequencies of absolute outlier rejection rules (78; 47.9%) are shown in Table 2, and frequencies of adaptive outlier rejection methods (63; 38.7%) are shown in Table 3.

**Table 2:** Frequencies of upper and lower RT cutoffs in the reviewed literature

Outlier definition	<100 ms		<150 ms		<200 ms		<250 ms		<300 ms		<350 ms		*None*		*Total*	
	n	%	n	%	n	%	n	%	n	%	n	%	n	%	n	%
>1000 ms			3	1.84%											3	1.84%
>1500 ms	2	1.23%	6	3.68%					1	0.61%	6	3.68%	6	3.68%	21	12.88%
>1700 ms													1	0.61%	1	0.61%
>2000 ms	1	0.61%			20	12.27%	1	0.61%	4	2.45%			7	4.29%	33	20.25%
>3000 ms									3	1.84%			2	1.23%	5	3.07%
>3500 ms													1	0.61%	1	0.61%
>4000 ms									1	0.61%					1	0.61%
>5000 ms					1	0.61%									1	0.61%
>10,000 ms									2	1.23%			1	0.61%	3	1.84%
*None*	1	0.61%	3	1.84%	2	1.23%	1	0.61%					87	53.37%	94	57.67%
*Total*	4	2.45%	12	7.36%	23	14.11%	2	1.23%	11	6.75%	6	3.68%	105	64.42%	163	100%

**Table 3:** Frequencies of adaptive outlier rejection methods in the reviewed literature

Outlier definition	Both sides		Upper side	
	N	%	N	%
Upper and/or lower 1%	10	6.13%		
Upper and/or lower 2%	1	0.61%		
1.5 SD			1	0.61%
2 SD	3	1.84%	2	1.23%
2.5 SD	7	4.29%	1	0.61%
3 SD	28	17.18%	7	4.29%
Multiple methods	1	0.61%		
*Total*	50	30.67%	11	6.75%
*Unclear*	2	1.23%		
*None*	100			

#### Error rules

In most experiments, the authors excluded error trials (115; 70.55%), while others either included them (34; 20.86%), replaced them with a block mean RT of correct trials plus a penalty (7; 4.29%), or required participants to give correct responses to complete the trial (7; 4.29%).

#### Bias score algorithms

We categorized the observed bias score algorithms where possible, and gave them systematic names, which will be used in the remainder of the article. They are shown in Table 4.

**Table 4:** Bias score algorithms and how frequently they have been used

Name	N	%	Formula
Median category-specific difference	47	28.83%	${\overset{\sim }{RT}}_{avoid\ target}-{\overset{\sim }{RT}}_{approach\ target}$
Mean category-specific difference	32	19.63%	${\overline{RT}}_{avoid\ target}-{\overline{RT}}_{approach\ target}$
Category-specific difference D-score	6	3.68%	$\frac{{\overline{RT}}_{avoid\ target}-{\overline{RT}}_{approach\ target}}{SD_{target\ RT}}$
Double median difference	18	11.04%	$\left({\overset{\sim }{RT}}_{avoid\ target}-{\overset{\sim }{RT}}_{approach\ target}\right)-\left({\overset{\sim }{RT}}_{avoid\ control}-{\overset{\sim }{RT}}_{approach\ control}\right)$
Double mean difference	3	1.84%	$\left({\overline{RT}}_{avoid\ target}-{\overline{RT}}_{approach\ target}\right)-\left({\overline{RT}}_{avoid\ control}-{\overline{RT}}_{approach\ control}\right)$
Median compatibility score	4	2.45%	${\overset{\sim }{RT}}_{avoid\ target\ or\ approach\ control}-{\overset{\sim }{RT}}_{approach\ target\ or\ avoid\ control}$
Mean compatibility score	4	2.45%	${\overline{RT}}_{avoid\ target\ or\ approach\ control}-{\overline{RT}}_{approach\ target\ or\ avoid\ control}$
Compatibility D-score	1	0.61%	$$\frac{\begin{array}{c}{\overline{RT}}_{avoid\ target\ or\ approach\ control}\\ {}-{\overline{RT}}_{approach\ target\ or\ avoid\ control}\end{array}}{SD_{RT}}$$
Median movement-specific difference scores	4	2.45%	${\overset{\sim }{RT}}_{avoid\ target}-{\overset{\sim }{RT}}_{avoid\ control}$ and ${\overset{\sim }{RT}}_{approach\ target}-{\overset{\sim }{RT}}_{approach\ control}$
Mean movement-specific difference scores	3	1.84%	${\overline{RT}}_{avoid\ target}-{\overline{RT}}_{avoid\ control}$ and ${\overline{RT}}_{approach\ target}-{\overline{RT}}_{approach\ control}$
*Multiple*	9	5.52%	
*Other*	3	1.84%	
*None*	20	12.27%	
*Unclear*	9	5.52%	
*Total*	163	100%	

#### Participant rejection rules

It was uncommon for participants to be rejected based on bad performance (42; 25.8%), but it is unclear whether this is because participants performed well in most studies, or their performance simply was not examined. If participants were rejected, it was most commonly on the basis of error rates (34; 20.9%), with an error rate above 25% being the most common cutoff (12; 7.36%), followed by error rates of 35% (6; 3.68%), and 20% (4; 2.45%). Less often, participants were rejected because they had RTs that were too slow (6; 3.68%), or because they had too few trials remaining after error and outlier removal combined (4; 2.45%). In a minority of studies, participants were rejected not (only) due to high error rates or slow RTs, but (also) because their bias scores were too outlying (11; 6.75%), their scores had too much influence on the regression outcome (1; 0.61%), or for unclear reasons relating to the magnitude of their scores (1; 0.61%). Many of the examined experiments gave the impression that no participant rejection rule was defined beforehand, but participants were rejected following data inspection.

#### Pipelines

We empirically observed a total of 108 unique pre-processing pipelines across 163 studies, out of 218,400 possible combinations, computed by multiplying the numbers of all unique observed pre-processing methods at each step with each other.

### Discussion

We found that some pre-processing methods were quite common (e.g. excluding trials deviating more than 3 SD from the participant mean), but there is still much heterogeneity in the literature, as only a few studies used identical pre-processing methods, which makes it difficult to discern whether divergent results in the literature are due to differences in experimental design, pre-processing, or chance. In the following discussion of Study 1, we will review the observed and hypothetical new pre-processing decisions based on methodological considerations, in anticipation of Studies 2, 3, and 4.

#### Outlier rejection

Various methods are used to flag and remove implausible or extreme RTs. This is especially important considering that non-robust statistics are much more strongly influenced by individual extreme outliers than by a multitude of regular RTs; as such, outliers inflate type I and II error rates by suppressing effects that exist and creating effects that do not exist in real life (Dixon, 1953; Ratcliff, 1993).

##### Fixed RT cutoffs

It seems sensible to remove outliers based on a cutoff that is adapted to the specific study but fixed across participants in that study. This is based on the reasoning that there is a high likelihood that RTs above or below certain values have a different origin than the mental process being measured (Ratcliff, 1993). The removal of such RTs is thus thought to enhance the validity of the data. For example, when a participant forgets the instructions and tries to remember them, this can result in a 4-second RT caused by memory search rather than by stimulus recognition and decision-making. The same goes for fast outliers: it is known that people only begin to recognize a stimulus after about 150 ms, and they only begin giving above-chance responses from 300 ms and onwards (Fabre-Thorpe, 2011). Given this, a 50 ms RT is most likely not related to the stimulus that has just been shown on the screen. It remains unclear, however, what the ideal cutoffs are. Ratcliff (1993) found that a RT cutoff of 1500 ms led to results with decent power to detect a group difference, when said group difference was in the mean of the distribution, but also when it was in the tail of the distribution instead. This study, however, utilized simulated data that may not correspond with effects observed in real life. Further complexity is introduced by the fact that some stimuli are more visually or conceptually complex than others and may thus require more processing time before the participant is capable of responding correctly using the cognitive mechanism under study.

##### Means and SDs

By far the most common adaptive outlier rejection method is to removes all RTs that deviate more than 3 SD from the participant’s mean. Ratcliff (1993) found that very strict SD boundaries (e.g. M + 1.5 SD) reasonably salvage power to detect group differences when the group difference is in the group means, but significantly weaken power when groups primarily differ in the length of the right tail of the distribution; this suggests that the benefit of using means and SDs can depend on the nature of the task. Both means and SDs are themselves also highly influenced by outliers. Thus, when using this method, extreme outliers widen the SD and mask smaller outliers that would otherwise have been detected after an initial exclusion of extreme outliers. Additionally, means and SDs are only correct descriptors for symmetric distributions, which RTs are not; as such, this method is prone to removing only slow outliers, while ignoring extremely fast RTs that technically do not deviate more than 3 SD from the mean, but are nevertheless theoretically implausible. Hence, this method is often combined with absolute outlier cutoffs that eliminate extreme RTs at both ends of the distribution before means and SDs are computed for further outlier rejection.

Alternatively, one may turn to robust estimators of the mean and SD, such as the median and the median absolute deviation (MAD, (Hampel, 1985)), respectively. Unlike the mean, the median is not disproportionately affected by outliers compared to non-outlying datapoints, as the median assigns equal leverage to every data point. Hence, it is not affected by the outliers’ extremeness, but only by their count. Similarly, the MAD is a robust alternative to the SD, calculated by computing the deviation of all data points from the median, removing the sign from these values, computing their median, and multiplying this value by a constant to approximate the value that the SD would take on in a normal distribution.

##### Percentiles

One of the less common ways to deal with outliers was to remove a fixed percentage of the fastest and slowest RTs from the data (10 out of 163 studies). This method has the advantage of not ignoring fast outliers. However, it is independent of the characteristics of the RT distributions under investigation, and is thus likely to remove either too few or too many outliers, depending on the data.

##### Outlier tests

While much less common than any of the aforementioned methods, another method that deserves mention is the significance testing of outliers. The Grubbs test (Grubbs, 1950) was used by e.g. Saraiva et al. (2013), among others, to detect whether the highest or lowest value in the data significantly deviates from the distribution. When found to be significantly different, this value is removed, and the process is repeated until no more significant outliers are detected.

### Error handling

Study 1 revealed four ways of dealing with error trials: including them in the analyses, excluding them, replacing them with the block mean plus a penalty, or requiring the participant to give a correct response during the task itself and defining the RT as the time from stimulus onset until the correct response. Which method is ultimately the best depends on whether error trial RTs contain information on approach–avoidance bias. After all, some implicit tasks are based entirely on errors (e.g. Payne, 2001). Raw error counts sometimes show approach–avoidance bias effects (Ernst et al., 2013; Gračanin et al., 2018; van Peer et al., 2007) but often they do not (Glashouwer et al., 2020; Heuer et al., 2007; Kahveci et al., 2021; Neimeijer et al., 2019; Radke et al., 2017; von Borries et al., 2012), and it is unclear why some studies find such an effect while others do not. Therefore, we will examine how the different types of error handling affect reliability and validity (Study 4).

### Bias score computation algorithms

#### Category-specific and movement-specific difference scores

The category-specific difference score is the most popular bias scoring algorithm (93 in 163 studies). To compute it, one subtracts aggregated approach RTs from aggregated avoidance RTs for a single stimulus category (Table 1: quadrant A minus B, or C minus D). Movement-specific difference scores are less popular, but they similarly contrast a single condition to another, in this case by subtracting the approach or avoidance RT for a target stimulus from the approach or avoidance RT of a control stimulus. In the resulting score, positive values imply that the target stimulus is approached or avoided faster than the control stimulus (Table 1: quadrant C minus A, and D minus B; note that the Table displays these the other way around). As we discussed in the introduction, these scores are problematic when interpreted on their own, because they do not account for interpersonal and overall differences in how fast participants perform approach and avoidance movements, and how fast they classify the stimuli into their categories, respectively. This contamination with motor or classification effects can produce bias scores with extremely high reliabilities that do not correlate with any relevant interpersonal metric, because the difference score consists primarily of contaminant rather than stimulus-related approach–avoidance bias (as found by e.g. Kahveci, Meule, et al., 2020). To hold any meaning, they need to be contrasted with their opposite equivalent, i.e. approach scores with avoid scores, and target stimuli with control stimuli. This can be done through subtraction or by comparing the two scores in an analysis, such as ANOVA. Therefore, we primarily focus on double-difference scores in this article.

#### Double-difference scores

Double-difference scores cancel out effects other than stimulus category-specific approach–avoidance bias, by subtracting approach–avoidance scores for a control or comparison stimulus category from those of a target stimulus category (Table 1: quadrants [A-B]-[C-D]). They represent the advantage of approaching over avoiding one stimulus category relative to another. We will examine how mean-based and median-based double-difference algorithms compare in their ability to recover reliable and valid approach–avoidance bias scores.

#### Compatibility scores

These scores involve *averaging* all RTs from the bias-compatible conditions together, and subtracting this from the average of all RTs in the bias-incompatible conditions taken together. When one measures approach–avoidance bias towards palatable food, for example, the bias-compatible conditions involve approaching food and avoiding control stimuli (quadrants B and C of Table 1), while the bias-incompatible conditions involve avoiding food and approaching control stimuli (quadrants A and D of Table 1). When there is an equal number of trials in each of the four conditions, compatibility scores (e.g. $\overline{avoid\ target\ or\ approach\ control}-\overline{approach\ target\ or\ avoid\ control}$) are thus functionally identical (though halved in size) to double-difference scores (which can be reformulated as $\left(\overline{avoid\ target}+\overline{approach\ control}\right)-\left(\overline{approach\ target}+\overline{avoid\ control}\right)$. However, when there is an unequal number of trials in the conditions contained within the averages, the condition with more trials has a larger influence on the average than the condition with fewer trials, which can reintroduce RT influences that a double-difference score is meant to account for, such as stimulus-independent differences in approach–avoidance speed. Imagine, for example, that a participant particularly struggled with avoiding palatable food stimuli, and made a disproportionate number of errors in this particular condition. After error exclusion, their final dataset thus contains 20 avoid-food trials and 40 trials of each other condition. The mean RT of the incompatible conditions is thus more strongly influenced by the 40 approach-control trials than by the 20 avoid-food trials and it fails to cancel out the stimulus-independent difference between approach and avoid trials. Therefore, it is almost always an impure measure of approach–avoidance bias, as we will show in a further analysis.

#### The D-score correction

The D-score correction controls for the fact that larger differences between conditions emerge when a participant has a wider RT distribution, which occurs when they respond more slowly, as demonstrated by Wagenmakers and Brown (2007). The D-score was introduced by Greenwald et al. (2003) for the Implicit Association Task and was also adopted in AAT research (Wiers et al., 2011). Many different types of D-scores were reported in the AAT literature, with the common thread being that a mean-based difference score of some kind is divided by the SD of the participant’s RTs. It makes sense to cancel out the effect of narrower or wider SDs, as these can be caused by a myriad of causes other than underlying approach–avoidance bias, such as age, fatigue, and speed-accuracy trade-offs. However, this slowing cannot be entirely disentangled from the slower responding that may occur when individuals have more difficulty performing the task due to a strong and rigid approach–avoidance bias. Hence, it is as of yet unclear whether the D-score correction helps or hurts the validity of the AAT and will therefore be examined here.

#### Multilevel random effects

This scoring method has recently been introduced by Zech et al. (2020). It involves fitting a mixed model and extracting the by-participant random slopes representing the desired contrast between conditions. For example, a contrast between approach and avoidance can be retrieved by extracting the random effect of movement direction (0 = avoid, 1 = approach), and a double-difference score can be retrieved by extracting the interaction between movement direction and stimulus category (0 = control, 1 = target). This method allows for the inclusion of known covariates influencing individual RTs such as trial number, temporal proximity of error trials, and individual stimulus recognition speeds. Due to its novelty and good performance in the aforementioned study, we included this approach here and chose to examine it in the following analyses.

## Study 2: Susceptibility of compatibility scores to confounding caused by differences in trial count between conditions

### Introduction and method

As mentioned, compatibility scores are a problematic measure of approach–avoidance bias when the number of trials in each condition is unequal, which is bound to be the case when outliers and error trials are removed. We demonstrated this by simulating AAT datasets and examining how reliability is impacted by the removal of trials from one specific condition.

#### Examined methods

We examined double-difference and compatibility score variants of the four archetypal data aggregation methods described in Study 1: means, medians, D-scores, and multilevel random effects. The formulas for the first three of these methods are described in Table 1. As for the multilevel methods, multilevel double-difference scores were computed by extracting the per-participant random effect coefficients of a movement × stimulus-type interaction (computed in R with the formula RT ~ movement_direction * stimulus_category + (movement_direction * stimulus_category | Subject)), whereas multilevel compatibility scores were computed by extracting per-participant random coefficients of a main effect of stimulus-to-movement congruence (computed in R with the formula *RT ~ congruence + (congruence | Subject)*).

#### Dataset simulation

We simulated AAT datasets to produce values distributed with a right skew similarly to real AAT data and with adjustable differences between different conditions and between subjects. For each participant, we first randomly generated the mean RT, SD, movement direction RT difference, stimulus category RT difference, and bias effect RT difference (the true bias score), based on a predetermined sample-wide mean and SD for each parameter. After this, we generated gamma-distributed RTs whose means and SDs were shifted such that they matched predetermined parameters of their respective condition and participant. To be able to generate data with similar properties to those of real studies, we used means and SDs of the aforementioned parameters from the relevant-feature AAT described by Lender et al. (2018) with errors and outliers (RT <200 or RT >2000) removed; these parameters are described in Appendix 1. Each dataset featured 36 participants, each having 256 trials divided into four conditions. This data simulation procedure is available through the function *aat_simulate()* in the *AATtools* package (Kahveci, 2020) for R (R Core Team, 2020).

#### Analysis procedure

We simulated 1000 datasets based on the properties from Lender et al. (2018). We also simulated 1000 datasets where the RT difference between approach and avoid trials was doubled, as we hypothesized that unequal trial count is especially problematic for compatibility scores when RT differences between approach and avoidance trials are large. In each dataset, we removed one trial per participant from the approach-target condition (removing trials from any of the other condition instead should lead to identical effects on the compatibility score). After this, we computed double-difference scores and compatibility scores from the data using the aforementioned four archetypal data aggregation methods. We repeated this procedure of trial removal and score computation until 16 trials remained per participant.

#### Outcome measures

We evaluated the accuracy of the bias scores by correlating them with the predetermined true score on which the participants’ data were based. We refer to this measure as (true score) recoverability. We chose this measure, since it is intuitively easy to understand on its own, it is computationally much less costly than permutated split-half reliability, and it is equivalent to the square root of reliability, since

$$Cor\left(T,T+E\right)=\frac{Cov\left(T,T+E\right)}{\sqrt{Var(T)\cdot Var\left(T+E\right)}}=\frac{Cov\left(T,T\right)+ Cov\left(T,E\right)}{\sqrt{Var(T)}\cdot \sqrt{Var(T)+ Var(E)+2 Cov\left(T,E\right)}}=\frac{Var(T)}{\sqrt{Var(T)}\cdot \sqrt{Var(T)+ Var(E)}}=\frac{\sqrt{Var(T)}}{\sqrt{Var(T)+ Var(E)}}$$

where the Spearman-Brown-corrected split-half correlation is an estimator of

$$Cor\left(T+E,T+E\right)=\frac{Var(T)}{Var(T)+ Var(E)}$$

To be able to compare double-difference and compatibility scores, we also computed the probability that a randomly drawn double-difference score would be better than a randomly drawn compatibility score at recovering the true score. We arrived at this probability by computing the mean proportion of recoverability values of double-difference scores that were greater than recoverability values of each compatibility score. This was done separately for each aggregation method and number of missing trials.

### Results and discussion

**Fig. 1:**
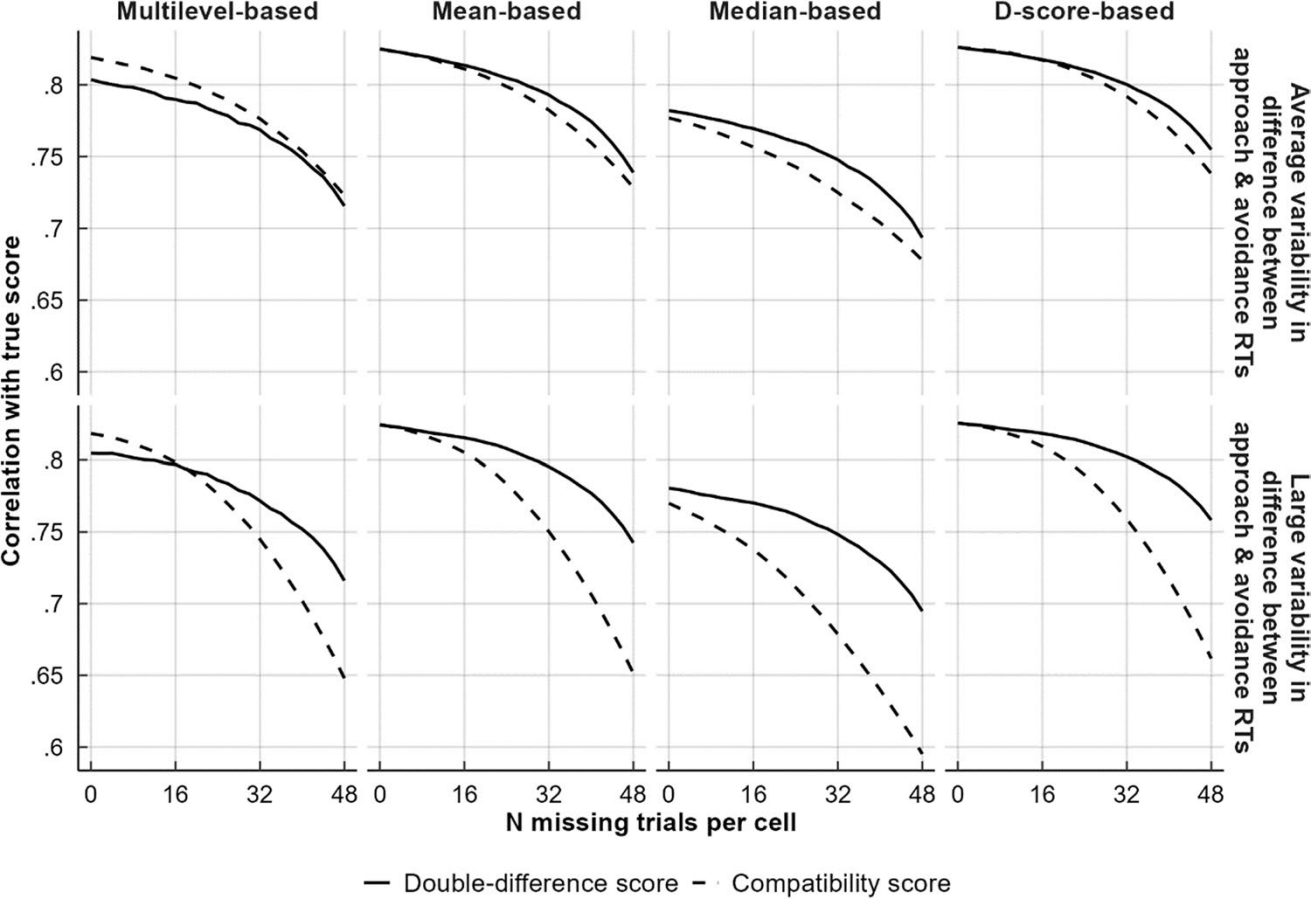
Effect of unequal trial count per condition on the recoverability of the true score from double-difference and compatibility scores that were based on means, medians, D-scores, and multilevel random effects

As depicted in Fig. 1, bias scores became increasingly inaccurate as the trial count became more unequal across conditions. This decrease in accuracy was larger for compatibility scores than for double-difference scores, and it was larger when there was more variability between the simulated participants in how much faster or slower they were to approach or avoid. Overall, the probability of a double-difference score being better than a compatibility score at recovering the true score was almost always above chance, being .79 at most. These probabilities are further depicted in Table 5.

**Table 5:** Probability of double-difference scores having higher true score recovery than compatibility scores

Variability in difference between approach and avoidance RTs	Aggregation method	Probability of double-difference scores having higher true score recovery than compatibility scores			
		0 missing trials	16 missing trials	32 missing trials	48 missing trials
Average	Multilevel-based	.46	.46	.49	.50
	Mean-based	.50	.51	.54	.54
	Median-based	.52	.55	.58	.55
	D-score-based	.50	.50	.54	.56
Large	Multilevel-based	.46	.50	.60	.69
	Mean-based	.50	.54	.66	.75
	Median-based	.54	.61	.71	.75
	D-score-based	.50	.54	.67	.79

Double-difference scores only performed worse than compatibility scores when computed using multilevel analysis, given either relatively small differences in trial count between conditions, or given average variability in the difference between approach and avoidance RTs. This contrast was driven by multilevel double-difference scores performing worse than their mean- and D-score-based counterparts, while multilevel compatibility scores performed on par. When bias scores were computed using medians, compatibility scores underperformed relative to double-difference scores even when trial counts were equal across conditions. In all other cases, the two scoring methods performed identically given equal trial counts across conditions, but diverged when these became unequal. Comparing the four score aggregation methods, D-scores best recovered the true score, and they were followed by mean-based scores, multilevel-based scores, and lastly, median-based scores.

Given the finding that compatibility scores either perform on par or worse than double-difference scores, we see little reason to use them when double-difference scores are available. We thus recommend using double-difference scores instead of compatibility scores, and we will do so ourselves in the remainder of the article. The only exception to these findings is in the case of multilevel-analysis-based scores, where compatibility scores were superior to double-difference scores unless groups were unequal in size and simultaneously had a large difference between approach and avoidance RTs. We will report on multilevel compatibility scores.

## Study 3: Simulation study of outlier rejection and scoring algorithms

### Introduction and methods

Given the heterogeneity in the literature as revealed in Study 1, we chose to empirically examine the impact of outlier rejection methods and scoring algorithms on reliability using data simulation. We simulated datasets to be able to control the number of outliers in the data, and we applied every unique combination of outlier rejection method and scoring algorithm to these datasets. We compared the methods to each other in their ability to recover the true scores on which the simulated data were based.

#### Examined methods

The examined bias computation algorithms included the double mean difference score, double median difference score, double-difference D-score, and multilevel compatibility scores, as described in the previous studies. We examined a number of outlier detection methods. First, we examined the sample-wide removal of the slowest and/or fastest percentile of trials (1% / 99%), because it is a common method in the AAT literature; we examined the per-participant removal of RTs exceeding the mean by 3 SD (M ± 3 SD), because it is similarly common; we examined the per-participant removal of RTs exceeding the mean by 2 SD (M ± 2 SD), as a representative of the more strict SD-based outlier removal methods that is sufficiently different from the aforementioned 3 SD method such that its effects on the data will be more detectable; we examined per-participant removal of RTs exceeding the median by ± 3 MADs (median ± 3 MAD), to be able to contrast the common 3 SD method to its robust counterpart; we examined repeated outlier testing and removal using one- or two-sided Grubbs tests (Grubbs), to represent outlier removal methods based on statistical testing rather than boundaries calculated from the data; and lastly, we contrasted these methods to no outlier rejection (None). We did not examine absolute outlier cutoffs in this study, as we were concerned that these, unlike adaptive outlier rejection methods, were too sensitive to the arbitrary properties of our current simulation (such as the mean and SD of the RTs), and would hence require the manipulation of these properties as well, which falls outside the scope of this article.

#### Analysis procedure

We generated 1000 datasets in the same manner as Study 2, with each dataset having the same properties as the relevant-feature AAT study of Lender et al. (2018) with outliers and error trials *included*. These datasets each had 36 participants with 256 trials each, spread across 2×2 conditions. When examining category-specific difference scores, we excluded all trials pertaining to the control condition from the data; when examining double-difference scores, the full dataset was used. In each dataset, we iteratively replaced one random additional RT with a *slow* outlier (mean = μ_participant_ + 1200, SD = 400 ms, gamma-distributed, shape = 3) in every participant’s data, (i.e. first one outlier, then two, then three) after which we separately applied each combination of one outlier rejection method to *slow* RTs and one bias scoring algorithm to the data. This was done 32 times per dataset, until 12.5% of each participant’s data consisted of outliers. To obtain the reliability of these combinations, we utilized the same true score recoverability measure that we used in Study 2; that is, we computed the correlation between the computed bias scores and the true (double-difference) bias scores that the data was generated from. We thus obtained for each of the 1000 datasets the recoverability of the true score from bias scores that were computed with each combination of outlier rejection method and bias score algorithm, from data with 0 to 32 outliers per participant. These recoverability values were averaged across datasets to gain an overview of how recoverable the true score was through each combination of methods at each number of outliers.

This procedure was repeated in another 1000 datasets, except we iteratively replaced one random RT with a *fast* outlier (mean = μ_participant_ – 500 ms, SD = 50 ms, gamma-distributed, shape = 3) in each participant’s data and applied outlier rejection to *fast* RTs before we computed bias scores and recoverability. We also repeated the same process in another 1000 datasets where we iteratively replaced one random RT with a fast outlier *and* another with a slow outlier.

### Results and discussion

**Fig. 2:**
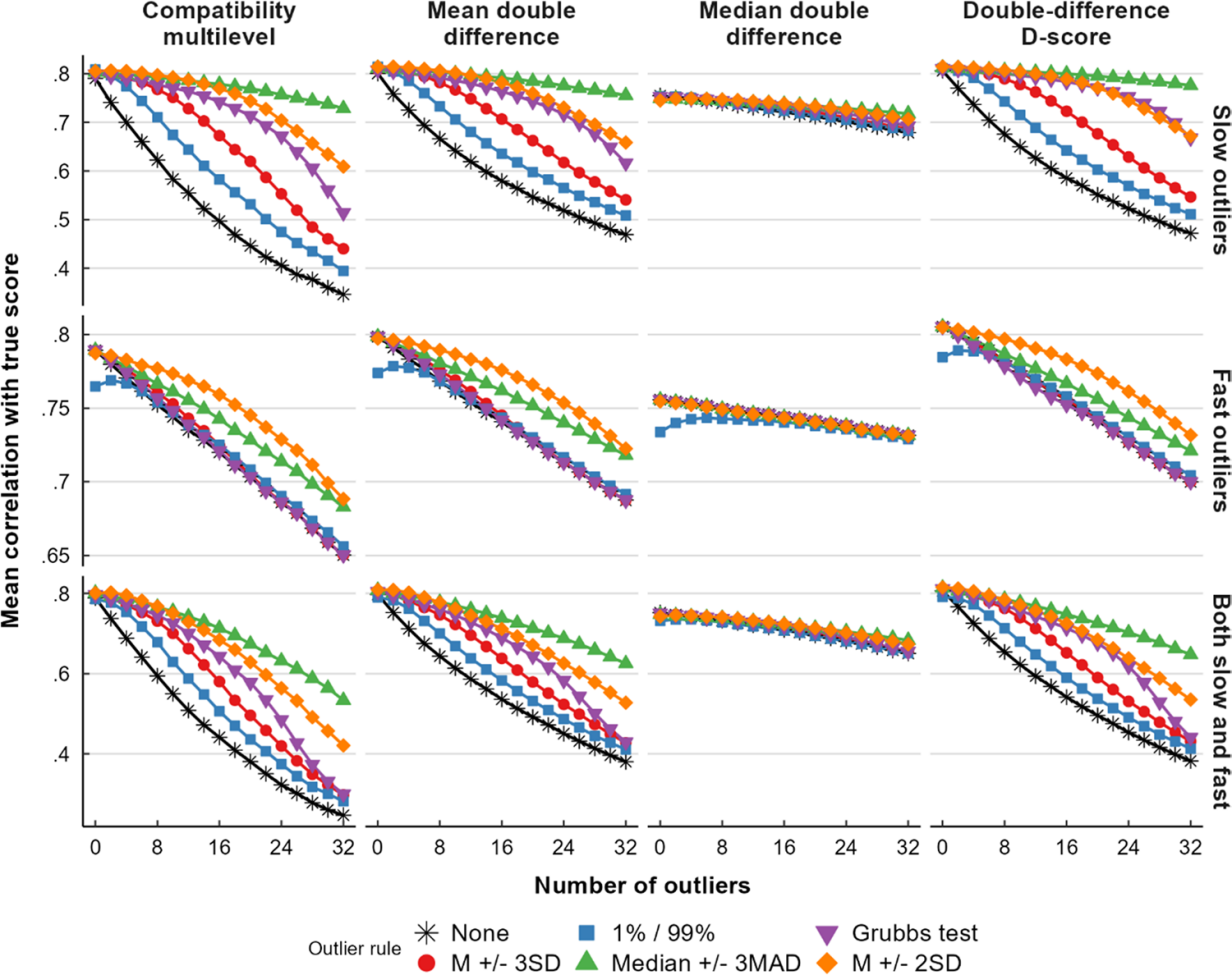
True score recoverability changes due to exclusion of outliers across the four double-difference scoring methods

In this section we report on results regarding the double-difference scores. Outcomes relating to category-specific difference scores were almost identical in pattern but lower in overall recoverability, and can be viewed in Appendix 2. We report on multilevel compatibility scores rather than multilevel double-difference scores since the former were better at recovering the true score in virtually all occasions. The results of the simulations for double-difference scores are depicted in Fig. 2. The sensitivity and specificity of the examined outlier rejection methods is also further discussed in Appendix 2. Correlations of bias scores with other aspects of the underlying data are also reported in Appendix 2. These reveal that multilevel bias scores are contaminated with variance from the participant mean RT. Whenever we report a correlation in this section, the associated number of outliers is reported as a subscript.

#### Slow outliers

The true score recoverability of the outlier rejection methods followed a similar pattern across all bias scoring algorithms. The true scores were most recoverable from bias scores computed using the median + 3 MAD method at almost any number of outliers (r_32_ = .70–.78). The mean + 2 SD method gave the second highest true score recoverability (r_32_ = .57–.71), but it was on par with the median + 3 MAD when there were 14 or fewer slow outliers per participant (2 SD and 3 MAD: r_12_ = .74–.80). Performing worse than these in terms of true score recoverability were Grubbs’ test (r_32_ = .48–.69), followed by the mean + 3 SD (r_32_ = .42–.69), percentiles (r_32_ = .38–.68), and lastly, no outlier removal (r_32_ = .33–.68). Percentile-based outlier rejection was effective when the data consisted of around 1% outliers (top percentile rejection: r_4_ = .75–.79, compared to no outlier rejection: r_4_ = .67–.75), but it failed to reduce the decline in true score recoverability compared to no outlier rejection when there were more outliers (from 4 to 32 outliers, true score recoverability for double mean difference scores went down by .37 for the percentile method and .35 for no outlier removal).

Comparing bias score algorithms, double median difference scores were barely influenced by outliers (r_0_ = .75 to r_32_ = .68 with no outlier removal) or outlier rejection. Double mean difference scores and double-difference D-scores were more strongly affected by outliers (means: r_0_ = .80 to r_32_ = .47, D-scores: r_0_ = .81 to r_32_ = .47 with no outlier removal), but they were better at recovering the true score when there were few outliers or when outliers were excluded with M + 2 SD or median + 3 MAD; across virtually all outlier rejection methods and numbers of outliers, D-scores were better than double mean difference scores at recovering the true score (correlations were, on average, .01 higher, up to .05). Multilevel compatibility scores showed the strongest decline in true score recoverability following the addition of outliers (r_0_ = .79 to r_32_ = .35 with no outlier removal), and outlier rejection failed to bring multilevel compatibility scores back on par with the other algorithms (e.g. when combined with 3 MAD outlier rejection, multilevel: r_32_ = .73, and D-score: r_32_ = .78). In addition, we report in Appendix 2 how the multilevel compatibility score also produces a much wider range of correlations with the true score than the other methods do, making it especially difficult to know whether any single application of this method will produce scores with the expected reliability; D-scores, in comparison, produced scores with the least variable correlation with the true score, indicating that this method is not only highly reliable but also consistently reliable. Overall, slow outliers strongly decreased true score recoverability (the reduction of recoverability from 0 to 32 outliers was between .03 and .44).

#### Fast outliers

Grubbs’ test and mean – 3 SDs almost completely failed to detect fast outliers, performing no better than no outlier rejection (Fig. 2). The best recoverability of the true score was obtained when classifying all RTs faster than 2 SDs below the mean as outliers, especially in data with many outliers (r_16_ = .73–.78). The median – 3 MAD method also led to better reliabilities (r_16_ = .72–.77) than no outlier rejection (r_16_ = .69–.76). Furthermore, compared to no outlier removal (r_0_ = .76–.81), removal of the fastest percentile of trials actually led to a *decline* in reliability which was especially noticeable when there were few or no fast outliers in the data (r_0_ = .73–.78).

Again, double median difference scores were only very slightly affected by outliers (r_0_ = .75 to r_32_ = .73), followed by D-scores (r_0_ = .81 to r_32_ = .70), double mean difference scores (r_0_ = .80 to r_32_ = .69), and lastly multilevel compatibility scores (r_0_ = .79 to r_32_ = .65); but median difference scores had lower reliability in the absence of outliers and never exceeded the reliability of D-scores when all trials more than 2 SD below the mean were removed. Overall, fast outliers had a relatively small influence on the reliability (the reduction of reliability from 0 to 32 outliers was between .02 and .14).

#### Bilateral outliers

Outlier rejection on data containing both slow and fast outliers led to results resembling a combination of the aforementioned findings, with the largest influence coming from slow outliers. Again, rejecting the top and bottom 1% of RTs reduced rather than improved the reliability when there were few to no outliers (percentile outlier removal: r_0_ = .73–.79; compared to no outlier removal: r_0_ = .75–.81). Bias scores were most reliable if outliers were removed by rejecting RTs deviating more than 3 MAD from the participant median (r_32_ = .50–.68), but with fewer outliers, reliabilities were on par when outliers were removed by rejecting RTs deviating more than 2 SD from the participant mean (2 SD: r_8_ = .74–.78; 3 MAD: r_8_ = .74–.79).

#### Conclusion

For outlier rejection methods, it can be concluded that percentile-based outlier detection removes too few outliers when there are many, and it removes too many outliers when there are few, to the point of making bias scores less accurate under common circumstances (e.g. Fig. 2, second row; lines with squares). Accordingly, percentile-based outlier exclusion appears to be disadvantageous. Given both slow and fast outliers, the remaining outlier rejection methods did not strongly differ in effectiveness when there were few outliers, but when there were many, median ± 3 MAD (Fig. 2, row 3; lines with upward triangles) outperformed mean ± 2 SD, which in turn outperformed Grubbs’ test and mean ± 3 SD (Fig. 2, row 3; diamonds, downward triangles, and circles). Mean ± 3 SD and Grubbs’ test also failed to reject most fast outliers (Fig. 2, row 2, circles and downward triangles), which suggests there is little point to using these methods to remove fast outliers; one should thus combine these two methods with an absolute outlier cutoff like, for instance, 200 ms.

Among the algorithms, double-difference D-scores and double mean difference scores were most reliable when there were few outliers – in data with many slow and fast outliers (>8%), they were outclassed by double median difference scores despite outlier rejection. Multilevel compatibility scores were less reliable than the aforementioned methods when there were no outliers, they became more unreliable when there were more outliers in the data, and their reliabilities were more inconsistent than those of other methods. Worryingly, applying outlier rejection was not enough to make multilevel compatibility scores as reliable as those derived with the methods not based on multilevel analysis. This casts doubt upon whether the use of this scoring method is justifiable. Median difference scores were shown to be nearly unaffected by outliers, but they were less reliable than the other methods when there were few outliers and outlier rejection was applied. Hence, it appears that the robustness of median-based scores may be outweighed by the reliability and consistency of mean-based scores and especially D-scores in conjunction with appropriate outlier rejection.

## Study 4: Comparison of validity and reliability of pre-processing pipelines on real data

### Introduction and methods

We next examined the effect of different pre-processing decisions on reliability and validity in six real datasets.

#### Description of the examined datasets and their criterion validity measures

We selected datasets to cover appetitive and aversive stimulus categories, relevant- and irrelevant-feature task instructions, and joystick and touchscreen input, to get results that can generalize to a wide range of future AAT studies. Datasets were only eligible if they measured both an initiation RT and a full motion RT, if they featured a target and control category, and if their bias scores were significantly correlated with a criterion variable. Properties of the datasets, such as mean RT and error rate, are shown in Appendix 2.

##### Datasets for “Erotica”

We used data from a single experiment fully described in Kahveci, Van Bockstaele, et al. (2020). In short, 63 men performed an AAT featuring eight blocks with 40 trials each. In four of these blocks, they had to classify images of women on the basis of whether the images were erotic or not (relevant-feature), and in the other four blocks they had to classify the images on the basis of hair color (irrelevant-feature). Half of the participants responded with the joystick and the other half using the keyboard. For analysis in the current study, five participants were removed: one with incomplete data, and four with a mean RT over 1000 ms. As the criterion validity measure, we chose the participants’ self-reported number of porn-viewing sessions per week, as we found that approach–avoidance scores correlated more strongly with this score than with other constructs measured in the study.

##### Datasets for “Foods”

We used data from a single experiment fully described in Lender et al. (2018). In short, 117 participants performed either of three joystick-AATs involving food and object stimuli where the correct movement direction was determined on the basis of different elements: stimulus content (*N* = 37), picture frame (*N* = 44), and a shape displayed in the middle of the stimulus (*N* = 36). Each task involved two blocks of 128 trials each. For the current study, we selected the content-based AAT as the relevant-feature task to be analyzed, and we selected the frame-based AAT as the irrelevant-feature AAT to be analyzed. For analysis in the current study, we removed one participant from the relevant-feature AAT with an error rate above 50%. As the criterion variable, we chose the restrictive eating scale (α = .90) of the Dutch Eating Behavior Questionnaire (van Strien et al., 1986), as we found that approach–avoidance bias scores correlated more strongly with this score than with other constructs measured in the study.

##### Datasets for “Spiders”

For the relevant-feature AAT involving spiders, we used data from a single study fully described in Van Alebeek et al. (2023). In short, 85 participants performed a relevant-feature AAT on a touchscreen where they were shown pictures of 16 spiders and 16 leaves, and were required to approach and avoid on the basis of stimulus content. Approaching involved sliding the hand towards the stimulus and then dragging it back to the screen center, while avoidance involved sliding the hand away from the stimulus. The task involved 128 trials divided into two blocks, and was embedded in a larger experiment which also included AATs involving butterflies, office articles, and edible and rotten food. As the criterion variable, we chose the Spider Anxiety Screening (α = .88; Rinck et al., 2002).

For the irrelevant-feature AAT involving spiders, we used data from a single study fully described in Rinck et al. (2021). In short, participants performed an irrelevant-feature go/no-go AAT on a touchscreen, where they were shown images of 16 spiders, 16 leaves, and 16 butterflies. Participants approached or avoided the spiders and leaves based on their position on the screen, while they were required not to respond to the butterflies. Responses always involved lifting the hand off the touchscreen and touching the stimulus, and then sliding it toward the other side of the screen. Thus, stimuli at the top of the screen were dragged closer and thus approached, while stimuli at the bottom of the screen were moved further away and thus avoided. After excluding all the no-go trials, the experiment consisted of 128 trials in a single block. The Spider Anxiety Screening was again used as criterion variable (α = .92).

#### Multiverse analysis

The six aforementioned datasets were pre-processed through many different pipelines, after which we computed the split-half reliability using the function *aat_splithalf()* in R package AATtools (Kahveci, 2020), as well as the criterion validity using Spearman correlations. We computed the average of 6000 random split-half correlations to obtain the randomized split-half reliability. We used 6000 iterations because we found in an analysis reported in Appendix 3 that, at most, 6000 random splits are needed to ensure that at least 95% of average split-half coefficients deviate less than .005 from the grand average of 100,000 splits. The examined components of the pipeline included the definition of the RT (initiation time, completion time), the lower RT limit (0 ms, 200 ms, 350 ms), the upper RT limit (1500 ms, 2000 ms, 10,000 ms), the adaptive outlier rule (none, mean ± 2 SD, mean ± 3 SD, median ± 3 MAD, <1% and >99%, Grubbs’ test), the error rule (keep errors, remove errors, replace errors with the block mean + 600 ms; further called error penalization), the algorithm type (category-specific difference, double-difference) and the algorithm aggregation method (mean difference, median difference, D-score, multilevel category-specific difference or compatibility). This led to a total of 2592 pipelines per dataset. The examined pipeline components were selected on the basis of their common use and methodological rigor as revealed by the literature review in Study 1, on the basis of results from the analyses in Studies 2 and 3, and with emphasis on newly (re)emerging methods in the field (e.g. Grubbs' test: Saraiva et al., 2013). In each analysis, the pre-processing steps were applied in the following order: the RT measure was selected, the lower and upper RT cutoffs were applied, error trials were excluded if required, outliers were excluded, error trials were penalized if required, and the bias scores were computed. During the computation of split-half reliability, participants were excluded from individual iterations if their bias score in either half deviated more than 3 SD from the sample mean of that half to ensure correlations were not driven by outliers.

#### Validity of category-specific difference scores

To gain an overview of the psychometric properties of category-specific difference scores, we performed a number of tests. We computed category-specific bias scores for target and control stimuli by subtracting participants’ median approach RT from their median avoid RT, both computed from initiation times of correct responses. We computed the correlation between bias scores for target and control stimuli. Across the whole of the multiverse analyses, we also computed the rank correlation between reliability and criterion validity per dataset and algorithm type (category-specific difference, double-difference). We excluded multilevel compatibility scores from analyses involving the irrelevant-feature AAT for reasons which are explained further in the results section.

#### Decision trees

Following the computation of reliability and criterion validity for each pipeline, we applied the Fisher *z*-transformation to the reliability and validity values to be able to analyze differences at both low and high levels of reliability and validity. We submitted the *z*-transformed reliabilities and validities as dependent variables to linear mixed decision tree analyses with random intercepts for dataset and fixed predictors for RT type, lower RT cutoff, upper RT cutoff, adaptive outlier rejection rule, error rule, and aggregation method. We used an alpha level of .001 and a maximum tree depth of 6 to prevent the decision trees from becoming too large to display. Decision trees were generated using R package glmertree (Fokkema et al., 2018). For display in plots and tables, the *z*-transformed correlations were averaged and then converted back to regular correlations.

### Results and discussion

#### Validity of category-specific difference scores

As can be seen in Table 6, target- and control-specific difference scores were positively correlated in all irrelevant-feature AATs, indicating that a significant portion of the variance in target-specific and control-specific stimuli is shared; this shared variance may originate from the interpersonal variability in participant’s overall approach–avoidance RT differences, as we speculated in Study 1. Conversely, there was a significant negative correlation for two of the three relevant-feature AATs, indicating that category-specific difference scores to target and control stimuli are related to a source of variance that increases one bias score but decreases the other, such as response slowdown between blocks.

**Table 6:** Correlations between target- and control-specific difference scores, and *t*-tests comparing control-specific bias scores to zero

		Correlation between category-specific difference scores of target and control stimuli		Correlation between reliability and criterion validity			
				Category-specific difference		Double-difference	
Instructions	Stimuli	*r*	*p*	*r*	*p*	*r*	*p*
Relevant-feature	Erotic	0	.988	.20	<.001	.31	<.001
	Food	−.40	.017	−.36	<.001	.15	<.001
	Spider	−.14	.189	−.20	<.001	.31	<.001
Irrelevant-feature	Erotic	.46	<.001	−.05	.152	−.01	.661
	Food	.38	.011	−.45	<.001	−.16	<.001
	Spider	.35	.001	.39	<.001	.08	.010

As reported in Table 6, when bias scores were computed with double-difference scores, reliability and criterion validity were positively correlated in four datasets, negatively in one, and not at all in one. When bias scores were computed with category-specific difference scores, reliability and criterion validity were negatively correlated in three studies, positively in two, and not at all in one. We expected positive correlations between reliability and criterion validity, as more reliable measures are less influenced by noise and could hypothetically capture the approach–avoidance bias more accurately, enabling stronger correlations with measures of similar constructs; negative correlations would imply that when bias scores become more reliable, they get better at measuring a construct that is different from implicit approach–avoidance bias; this would cast doubt upon the validity of the scores.

These findings thus err more towards supporting than rejecting the idea that category-specific difference scores run a risk of being contaminated with sources of variance unrelated to approach–avoidance bias of the target stimuli, and they run a higher risk than double-difference scores of becoming less valid as they become more reliable. In the remainder of this results section we will therefore report on double-difference scores, while results on category-specific difference scores can be gleaned in Appendix 4.

#### Variability in reliability and validity of different bias scoring algorithms

**Fig. 3:**
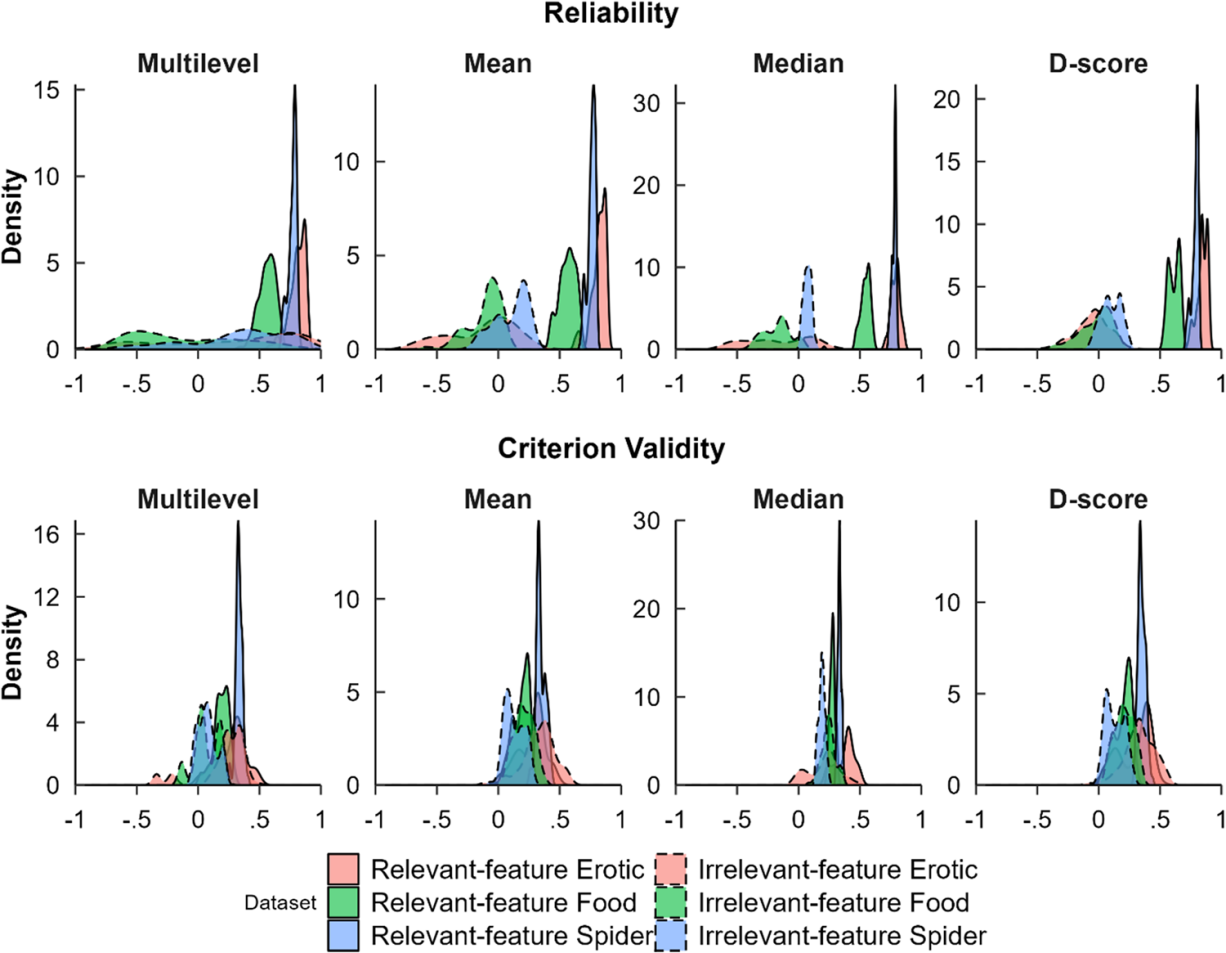
Distributions of reliability and validity coefficients acquired through different pre-processing pipelines in the six analyzed datasets. This figure depicts the distribution of reliability and criterion validity estimates from all different pre-processing pipelines. A wide distribution implies that differing pre-processing decisions had a large influence on the resulting reliability or criterion validity. Criterion validity is based on the correlation between approach–avoidance bias and a variable that was preselected on the basis of its significant correlation with approach–avoidance bias scores in that particular dataset. It is therefore of little value to focus on how high or low this value is in absolute terms. Rather, we guide the reader to focus on the spread or uncertainty of this value. In all cases, the validity of the irrelevant-feature AAT datasets is more spread out than that of the irrelevant-feature AATs

We sought to gain an overview of how much the various bias scoring algorithms are perturbed by other pre-processing decisions. Figure 3 and Table 7 depict the mean reliability and criterion validity of the various datasets, as well as several measures of spread. Criterion validity and especially reliability were found to strongly fluctuate depending on which pre-processing pipeline was used. Comparing task types, the irrelevant-feature AATs were, on average, less valid and much less reliable, and their reliabilities and validities were more strongly perturbed by pre-processing decisions. Variability of reliability estimates was especially strong in multilevel compatibility scores in the irrelevant-feature AAT, with extreme values reaching into the range of 1 as well as −1. This is likely due to the fact that small random effects are difficult to identify in multilevel models and can thus get contaminated with other aspects of the data such as the mean RT, as we demonstrated in Appendix 2. Hence, multilevel compatibility scores may not be valid for the irrelevant-feature AAT, as well as for any other task with very small effect sizes. Hence, we do not analyze multilevel compatibility scores in the irrelevant-feature AAT in the remainder of this article.

**Table 7:** Means, confidence intervals, and variability estimates for reliability and validity outcomes over all pipelines

AAT type	Algorithm	Reliability			Criterion validity		
		Mean *r*	95% CI	SD *z*	Mean *r*	95% CI	SD *z*
Relevant-feature	Multilevel	.74	.46, .88	.12	.27	.03, .45	.09
	Mean	.74	.45, .88	.12	.27	.05, .44	.08
	Median	.72	.49, .84	.07	.33	.23, .48	.06
	D-score	.77	.55, .89	.10	.28	.05, .45	.10
Irrelevant-feature	Multilevel	.23	−.67, .89	.60	.12	−.22, .38	.14
	Mean	−.05	−.61, .27	.21	.23	.03, .52	.11
	Median	−.09	−.56, .18	.18	.22	.01, .39	.09
	D-score	.01	−.32, .22	.12	.23	.03, .50	.10

*Note*: *SD*s represent the pooled SD f *z*-transformed, not raw, reliability and criterion validity estimates. Pooling was done by computing the variance within each dataset first and then averaging across datasets. Criterion validity is based on the correlation between approach–avoidance bias and a variable that was preselected on the basis of its significant correlation with approach–avoidance bias scores in that particular dataset. It is therefore of little value to focus on how high or low this value is in absolute terms. Rather, we guide the reader to focus on the spread or uncertainty of this value. In all cases, the validity of the irrelevant-feature AAT datasets is more spread out than that of the irrelevant-feature AATs

### Reliability decision trees

We used decision trees to deconstruct the complex nonlinear relationships between different factors in how they influence the reliability and validity of the six AATs.

**Fig. 4:**
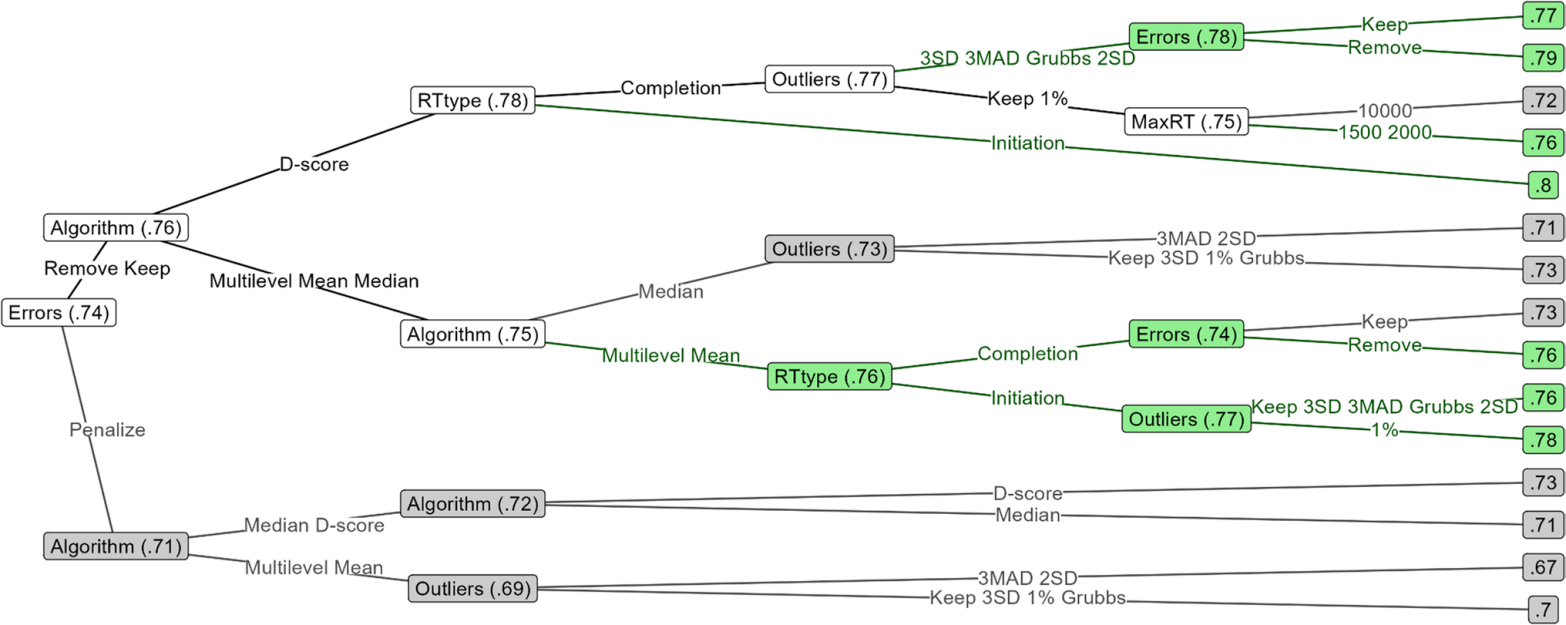
Decision tree of factors influencing the reliability of the relevant-feature AAT. The factors that the data was split by are denoted on each node, and the factor levels by which the data was split are depicted on the edges emerging from these nodes. The numbers displayed in each node represent the average reliability achieved by the decisions that led to that node. Particularly reliable and unreliable pathways are respectively depicted in green and grey

The reliability decision tree of the relevant-feature AATs is depicted in Fig. 4. The most influential decision was how to handle error trials: penalization (.71) gave worse reliability than error removal or retention (.76). The second most influential decision was algorithm: double-difference D-scores were the most reliable (.78) but could lead to lower reliability if lax outlier rules (upper RT limit of 10,000 ms, no adaptive outlier exclusion or percentile-based) were applied to completion times (.72); multilevel compatibility and double mean difference scores came in closely after (.76) and the only thing harming their reliability was retention (.73) rather than removal of errors (.76). Double median difference scores were the least reliable (.73) and were further harmed by the stricter outlier removal methods (.71; Median ± 3 MAD, M ± 2 SD).

**Fig. 5:**
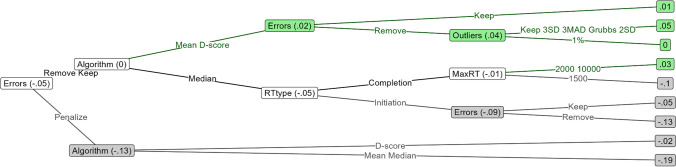
Decision tree of factors influencing reliability in the irrelevant-feature AAT. The factors that the data was split by are denoted on each node, and the factor levels by which the data was split are depicted on the edges emerging from these nodes. The numbers displayed in each node represent the average reliability achieved by the decisions that led to that node. Particularly reliable and unreliable pathways are respectively depicted in green and grey

The reliability decision tree of the irrelevant-feature AAT is depicted in Fig. 5. Reliability was very low for this task. Again, error penalization harmed reliability (−.13), though less for D-scores (−.02). Algorithm was the second most important decision when errors were not penalized: double median difference scores gave bad reliability (~ −.10), except with the use of completion times and less strict upper RT limits like 2000 ms or above (.03). Double mean difference scores and double-difference D-scores benefited the most from removal of error trials and from outlier handling with any method (.05) other than percentiles.

### Validity decision trees

We used the same methodology to construct decision trees for validity.

**Fig. 6:**
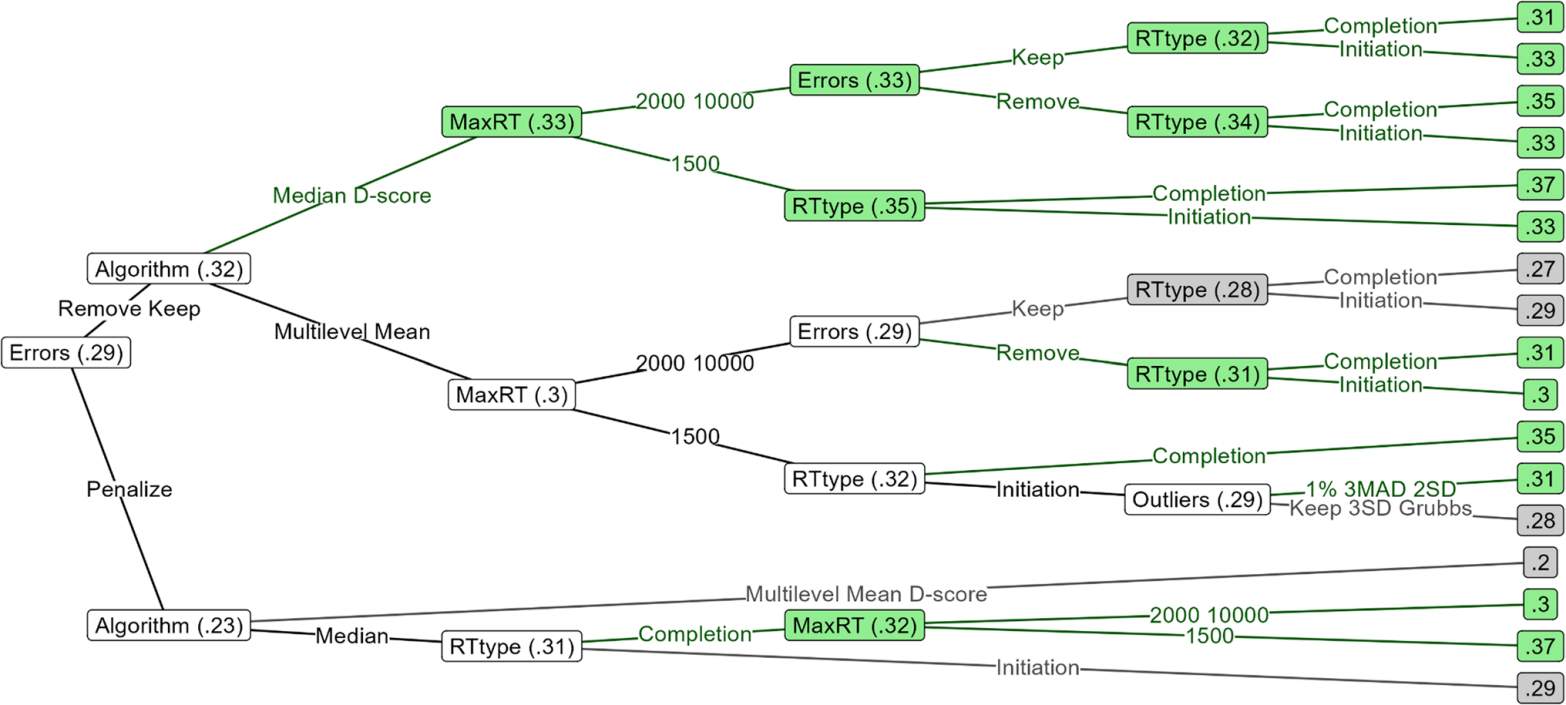
Decision tree of factors influencing the criterion validity of the relevant-feature AAT. The factors that the data was split by are denoted on each node, and the factor levels by which the data was split are depicted on the edges emerging from these nodes. The numbers displayed in each node represent the average criterion validity achieved by the decisions that led to that node. Particularly valid and invalid pathways are respectively depicted in green and grey

As depicted in Fig. 6, the criterion validity of the relevant-feature AAT was much less strongly perturbed by pre-processing decisions than its reliability was. Once again, error penalization was harmful to criterion validity on average (.23) compared to error removal and retention (.32). However, if error trial RTs were penalized, validity could be salvaged with the use of a combination of double median difference scores, completion times, and a 1500 ms RT cutoff (.37). When error trials were not penalized, validity was higher for double median difference scores and double-difference D-scores (.33) than for double mean difference scores or multilevel compatibility scores (.30). Additionally, validity often benefited slightly from removal of error trials, and subsequently, the use of completion times rather than initiation times.

**Fig. 7:**
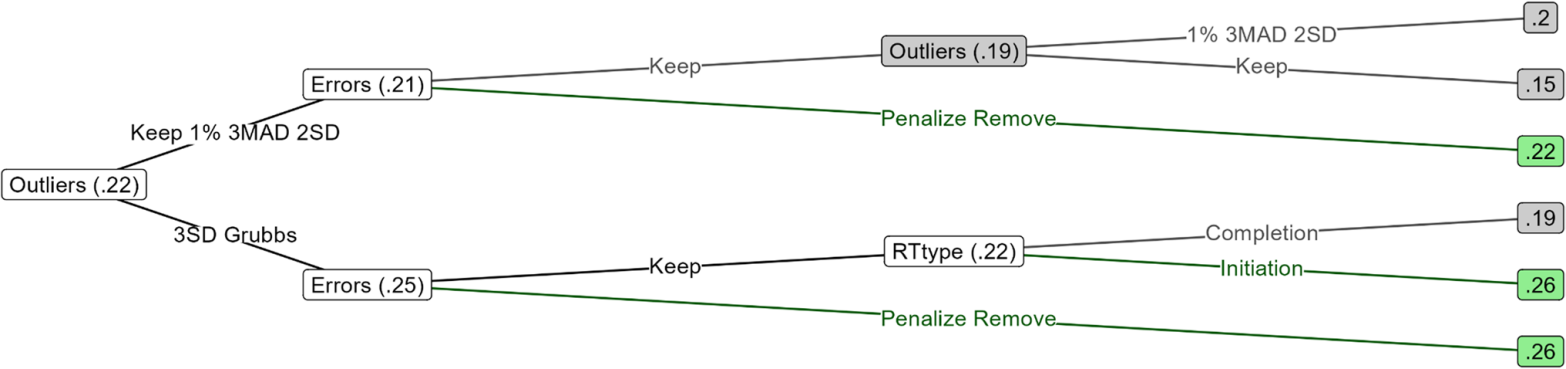
Decision tree of factors influencing the criterion validity of the irrelevant-feature AAT. The factors that the data was split by are denoted on each node, and the factor levels by which the data was split are depicted on the edges emerging from these nodes. The numbers displayed in each node represent the average criterion validity achieved by the decisions that led to that node. Particularly valid and invalid pathways are respectively depicted in green and grey

As depicted in Fig. 7, criterion validity outcomes were more ambiguous for the irrelevant-feature AAT. Criterion validity was higher with outlier rejection methods that were not strict and not lax, i.e. M ± 3 SD and the Grubbs test (.25); and validity could only be harmed within this branch by the combination of retaining error trials and using completion times (.19). With the other outlier rejection methods, reliability was best when error trials were removed or penalized (.22) rather than kept (.19). Unlike in every other decision tree, error penalization did not lower the outcome measure in this case.

## General discussion

There is a long chain of often arbitrary pre-processing decisions that researchers have to make before analyzing their data, and the wide variability in outcomes this can generate threatens replicability and scientific progress. Only recently have researchers begun to investigate the consequences of different decisions (Steegen et al., 2016), and a comprehensive study for the field of AAT research has so far been missing. We aimed to fill this gap here. Our selective literature review in Study 1 revealed a wide range of pre-processing practices in AAT studies. We subsequently used simulations (Studies 2 and 3) and analyses on real data (Study 4) to compare many of these practices, and obtained several findings that can inform further RT research. Importantly, we found large variability in the obtained reliability and validity outcomes depending on the chosen pre-processing pipeline. This highlights the fact that the varying practices do indeed muddy the waters of whether an effect is present or absent, and likewise, there is much to be gained from the informed choice of the study’s pre-processing pipeline. In turn, we will discuss the findings on error handling, outlier rejection, score computation, RT measurement, and instruction type. We will derive from these findings a set of recommendations, which are summarized in Table 8. We also consider implications for other RT-based implicit measures.

**Table 8:** Recommendations for pre-processing AAT data

	Less reliable/valid method	Methods with ambiguous outcomes	More reliable/valid alternative
Outliers	• Not rejecting outliers	• Removing the lowest and highest percentile of RTs sample-wide	• Rejecting RTs deviating more than 2 SD from the mean • Rejecting RTs deviating more than 3 SD from the mean • Rejecting RTs deemed outliers by repeated Grubbs’ tests • Rejecting RTs deviating more than 3 MADs from the median • Preceding aforementioned methods with the removal of RTs below and above reasonable fixed cutoffs*
Error trials	• Not removing error trials • Replacing error trials with the block mean plus a penalty		• Removing error trials
Bias score computation	• Compatibility scores • Multilevel double-difference scores • Category-specific multilevel scores in the irrelevant-feature AAT • Category-specific difference scores*	• Double median difference scores • Multilevel compatibility scores in the relevant-feature AAT	• Double-difference D-scores in conjunction with outlier rejection • Double mean difference scores in conjunction with outlier rejection

*Note:* Recommendations are displayed in order, with the worst (col. 1) and best (col. 3) methods displayed at the top of each list

* = primarily based on theoretical considerations

### Error trials

Most striking was the finding that replacing error RTs with the block mean RT plus a penalty (e.g. 600 ms) frequently led to lower reliability and validity. In Study 1 we found that this method was used in 7 out of 163 reviewed studies, likely due to the influence of the implicit association task literature in which this method is common. Furthermore, there was a smaller but noticeable disadvantage in reliability and validity when error trials were kept rather than removed, especially when trial completion times were used as the RT measure. Errors were kept in the data in 34 out of 163 reviewed studies.

### Outliers

Regarding RT cutoffs, we found that reliability and validity of real data were unaffected by the presence or absence of lower RT cutoffs; hence, this particular pre-processing decision may not strongly influence reliability and validity outcomes. Upper RT cutoffs did influence outcomes, though not that frequently. A cutoff of 1500 ms showed good validity for completion times in the relevant-feature AAT, while a cutoff of 1500 or 2000 ms showed slightly better reliability than a cutoff of 10,000 ms under very specific conditions. Despite this ambiguity, however, we do suggest that reasonably chosen lower and upper RT cutoffs be applied: both slow and fast outliers, however rare and insignificant they may be, still represent invalid data, and slow outliers still have a strong impact on subsequently applied adaptive outlier removal methods and RT aggregation.

We found no clear pattern regarding which outlier rejection method produced better results in real data; we did, however, find that removing outliers was better than not doing so. This contrasts with the results of our simulations, where true score recoverability followed a consistent pattern across outlier rejection methods from best to worst: median ± 3 MAD > mean ± 2 SD > repeated Grubbs tests > mean ± 3 SD > 1^st^ & 99^th^ percentiles > none. In our simulations, we found that dealing with outliers by rejecting the lowest and highest RT percentiles across the dataset can actually harm reliability, since this method does not distinguish between real outliers and regular RTs in very fast or slow individuals. It was used in 10 out of 163 reviewed studies. Furthermore, almost all fast outliers remained in our simulated data when we rejected RTs deviating more than 3 SDs from the individual mean or RTs that were significant outliers on Grubbs’ test, and hence, these methods should be used in conjunction with fixed cutoffs for fast outliers. Rejecting RTs deviating more than 2 SDs from the individual mean produced the best reliability outcomes in simulations involving fast or few slow outliers, but in real data the reliability and validity of this outlier rejection method often performed on equal footing with rejecting RTs deviating more than 3 SDs from the mean. Berger and Kiefer (2021) found in a series of simulation studies that SD-based outlier rejection, in contrast to MAD-based outlier rejection, is less prone to inflating type I error. Based on these findings and prior establishment of methods in the field, our preference thus goes towards either rejecting outliers deviating more than 2 SD from the mean, or towards rejecting RTs deviating more than 3 SDs after very fast slow RTs have been removed, as reported in Table 8.

We have two explanations for why the outcomes for outlier rejection in simulated and real data were divergent. First, the outlier rejection methods may have produced more divergent outcomes for our simulations simply because we simulated a large number of outliers: when the number of simulated outliers was smaller and more consistent with what occurs in real data (e.g. 4% of trials), the outlier rejection methods were much less distinguishable. Though less likely, an alternative explanation is that the simulation was based on incorrect assumptions. We assumed that RT differences between conditions are represented by shifts in the bulk of the RT distribution, rather than in the presence of more extreme RTs in one condition than in the other; depending on which of these two assumptions is used, results can be quite different, as demonstrated by (Ratcliff, 1993). This assumption may have favored outlier rejection methods that remove a larger number of extreme RTs, such as the MAD. More lenient outlier rejection methods would be favored if differences between conditions instead originated from differences in the number of extreme RTs. Future research should investigate whether RT differences between conditions in the AAT are represented by a larger number of extreme RTs or by shifts in the bulk of the RT distribution.

### Scoring algorithms

Regarding scoring algorithms, we reasoned that category-specific difference scores (approach stimuli – avoid stimuli) are confounded with stimulus-independent individual differences in approach–avoidance speed, and hence, these should always be contrasted with a reference stimulus category. In Study 4, we found that increasing the reliability of a category-specific difference score often decreases its validity, and that category-specific difference scores for target and control stimuli are positively correlated in the irrelevant-feature AAT, supporting the idea that these scores are contaminated, and become more contaminated when they are more reliable. We therefore opted to focus the majority of this article on double-difference scores. However, our concerns about category-specific difference scores need to be corroborated with more conclusive evidence in future empirical studies, which manipulate or track factors that differentially influence approach and avoidance RTs, such as posture, fatigue, muscle mass, and response labelling.

We demonstrated using simulations that compatibility scores become more inaccurate than double-difference scores when there is an unequal number of trials in different conditions, and they confer no benefits over double-difference scores; the only exception to this was in multilevel random effect scores. We found that multilevel random effects are inaccurate compared to other methods, and become increasingly inaccurate when bias scores are modelled with three model terms as in a double-difference score, rather than with a main effect, as in a compatibility score. Hence, double-difference scores should be preferred over compatibility scores except when bias scores are computed through multilevel modelling.

Among these, double-difference D-scores consistently had the highest validity and reliability and the lowest variability in outcomes, both in simulated and real data; we therefore express our clear preference for double-difference D-scores over the other methods. Double mean difference scores had more variable outcomes and were often slightly less reliable and valid.

We found in both simulations and real data—to our surprise—that double median difference scores led to lower reliability than double mean difference scores or double-difference D-scores in conjunction with adequate outlier rejection. Double median difference scores were only more reliable in simulated data with many outliers (>8%). In validity, there was not as much of a difference between algorithms so long as errors and outliers were removed. Hence, we draw no strong conclusions on whether double median difference scores are to be discouraged or not.

We found that multilevel compatibility scores were more strongly affected by outliers than any other scoring algorithm, and applying outlier rejection did not fully remedy this issue. Multilevel compatibility scores also had the largest unpredictability in outcomes both simulations and real data, and especially in the irrelevant-feature AAT. We hypothesize that this is due to the fact that it can be difficult for multilevel models to identify small random effects, as occur in the irrelevant-feature AAT, where bias scores explain only a small proportion of the RT variance. Hence, we recommend against using multilevel random effect scores in the irrelevant-feature AAT, and we remain ambivalent about their use in the relevant-feature AAT. Further research is needed to demonstrate whether this method has any advantages that make it preferable over the algorithms that do not use mixed modelling. In particular, multilevel modelling (or per-participant regression) could account for trial-level contaminants of RTs, such as post-error slowing, fatigue and learning effects, and stimulus-specific confounds such as recognition speed, or visuospatial complexity. It is as of yet unclear how exactly these contaminants could best be modelled, and whether their inclusion benefits the validity of the bias scores.

### RT definitions

Regarding RT definitions, our findings were somewhat inconclusive: the only consistent pattern was that completion times are less reliable and valid when error trials are also kept in the data. We therefore cannot draw any conclusions as to which of these two RT definitions is preferable. We suggest that the RT definition be chosen on the basis of theoretical considerations and previous research in a specific field. As we found in our own previous research with touchscreen-based AATs, approach–avoidance biases may express themselves primarily at the movement planning stage, such as when the target stimuli are foods (Kahveci et al., 2021; Van Alebeek et al., 2021), or during movement execution, such as when the target stimuli are spiders (Rinck et al., 2021). Since very few studies have explored the outcomes of multiple RT definitions (see also: Rotteveel & Phaf, 2004; Solarz, 1960), we recommend that this be done more often in future research.

### Limitations and future directions

We were unable to investigate on which basis to include or exclude participants from AAT studies, for example, on the basis of extreme mean RT, error rate, outlier count, or bias score relative to the rest of the sample. Such an investigation would require the analysis of far more datasets, and hence, this is to be addressed by future research. For now, it may be sensible to reject participants on the basis of preset criteria regarding error rates and mean RTs, as these can signal that the data of a particular participant do not sufficiently represent the mental process under study. Similarly, when non-robust analysis methods are used, outlying bias scores should be removed.

We did not investigate the impact of several less common pre-processing approaches that address problematic aspects of the data overlooked by most reviewed methods. RT transformations, such as square root, natural logarithm, and inverse transformations, can reduce the rightward skew of the RT distribution and thereby de-emphasize the influence of slow RTs on subsequently computed bias scores that are based on means or regression. Similarly, there are a number of outlier rejection methods that can deal with skewed distributions, such as the use of interquartile ranges, and exclusion using asymmetric SDs; these currently remain unexplored in this article and the wider AAT literature. RTs can also be excluded by their temporal position within the block, as participants are often still memorizing the instructions at the start of the block; hence, exclusion of trials at the start of the block is a recommended pre-processing step for the brief IAT (Nosek et al., 2014).

### Generalization to other RT paradigms

The current methodological findings cause concern for how data are analyzed in other RT tasks. However, it is difficult to forecast how the examined methods affect the validity of other tasks, as other tasks might depend on aspects of the data that are masked by this study’s recommendations. Ideally, the current multiverse decision tree methodology could be applied to every popular experimental paradigm to confirm whether it is beneficial or detrimental how these tasks are currently pre-processed. It is particularly important that such a multiverse analysis is performed on paradigms where the most commonly used pre-processing pipelines include methods we found to be detrimental. The IAT, for example, is commonly analyzed by penalizing error trials and including outliers in the data. These recommendations by Greenwald et al. (2003) were adopted in a minority of AAT studies that used the D-score (e.g. Ferentzi et al., 2018; Lindgren et al., 2015; Van Alebeek et al., 2021), and are contradicted by our findings.

This being said, a number of our findings are purely statistical in nature and can be expected to generalize regardless of the paradigm. We demonstrated in Study 2 that less accurate aggregated scores are obtained when averaging together two conditions with unequal trial counts (as in compatibility scores) instead of computing separate averages for each and adding those together (as in double-difference scores). This disadvantageous practice is common in research on the IAT (Greenwald et al., 2003). Additionally, rejecting the top and bottom 1% of RTs as outliers will also lead to the removal of an inappropriately low or high number of trials in other paradigms, although this method currently sees little use outside the AAT literature. Lastly, fast outliers will also remain undetected in other paradigms when most of the adaptive outlier rejection methods that we examined are applied.

Lastly, it remains to be explored further in the AAT and in other paradigms whether it is problematic to contrast two response conditions to target stimuli without further contrasting these to control stimuli, as with category-specific difference scores. Stimulus-independent biases favoring one response over the other are common and cannot always be prevented through good experimental design. It remains to be shown, however, how influential they truly are, especially when responses consist of mere button-presses rather than full-limb movements as with the joystick.

### Conclusions

Far from delivering a one-size-fits-all recommendation for pre-processing the AAT, our review, simulations, and multiverse decision tree analyses have recovered a number of more reliable and valid methods, while eliminating a smaller number of methodologically harmful “forking paths” in the garden of AAT pre-processing decisions, as shown in Table 8. As some of these harmful practices are highly common (e.g. error trial retention or penalization) or even dominate the field (e.g. median category-specific difference scores), we hope that the recommendations of the current study will help to significantly improve the overall reliability and validity of future AAT studies.

## Figures and Tables

**Fig. 8:**
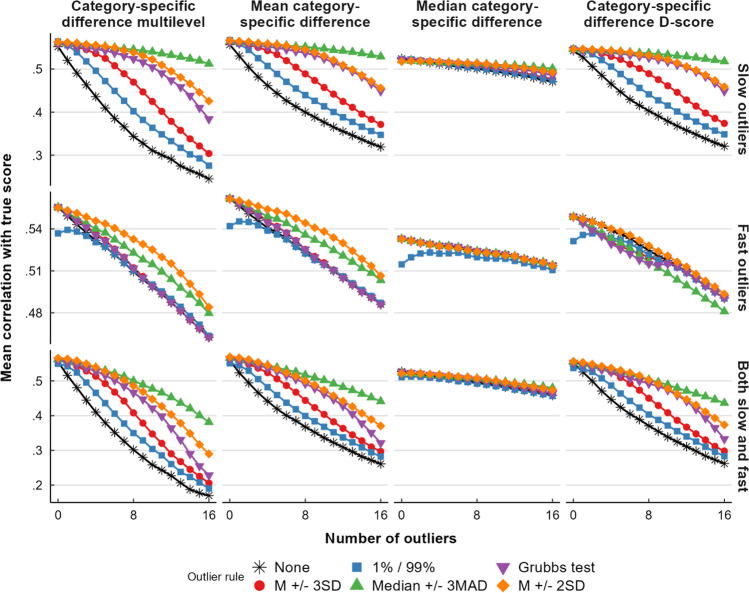
True score recoverability changes due to exclusion of outliers across the four category-specific difference score methods

**Fig. 9:**
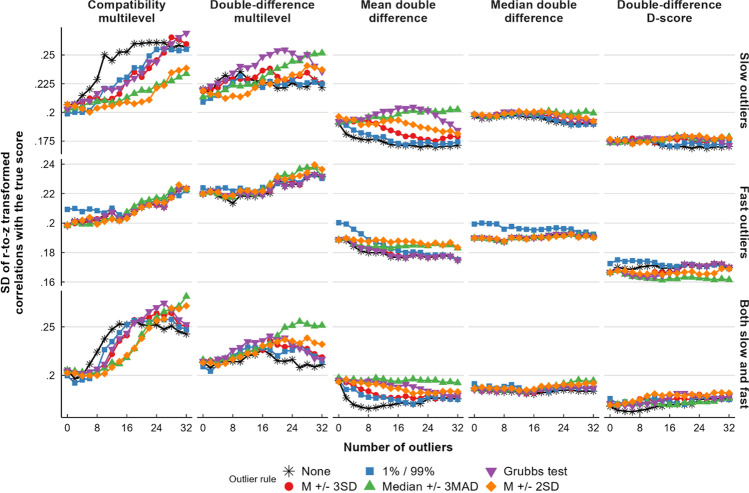
Variability of true score recoverability computed with different outlier rejection methods and scoring algorithms at different numbers of outliers

**Fig. 10:**
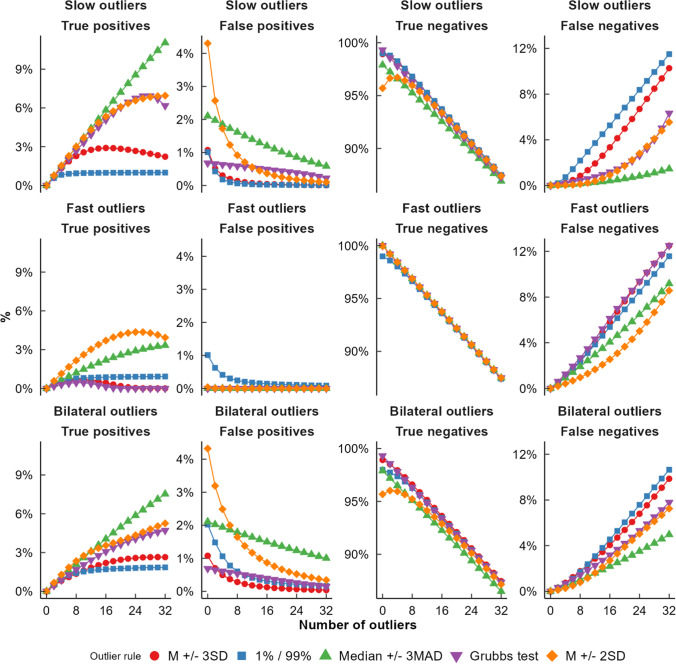
Outlier detection rates for different detection methods and types of outliers

**Fig. 11:**
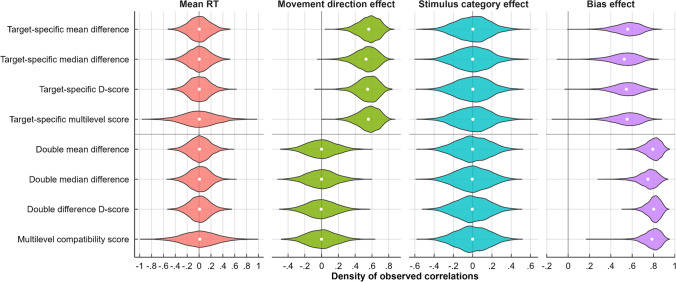
Correlations of bias scores with parameters used to generate the data

**Fig. 12:**
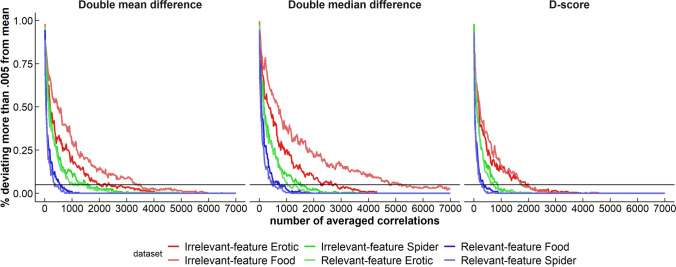
Percentage of pooled split-half correlations deviating more than .005 from the grand average as a function of the number of split-half correlations included in the pool

**Fig. 13:**
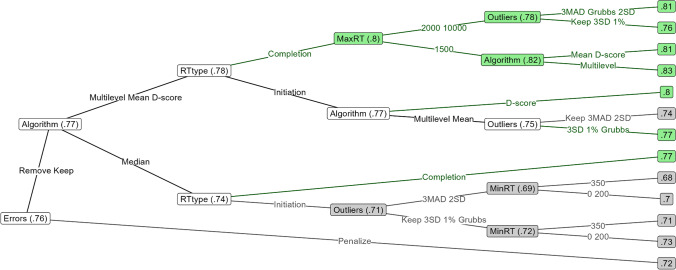
Decision tree of factors influencing the reliability of the relevant-feature AAT, as computed with category-specific difference scores

**Fig. 14:**
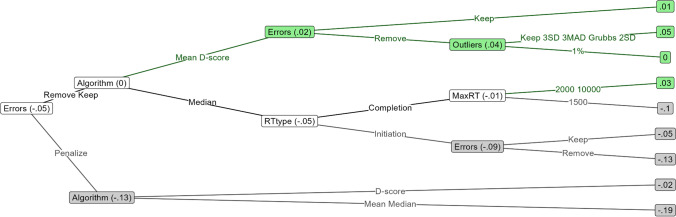
Decision tree of factors influencing the reliability of the irrelevant-feature AAT, as computed with category-specific difference scores

**Fig. 15:**
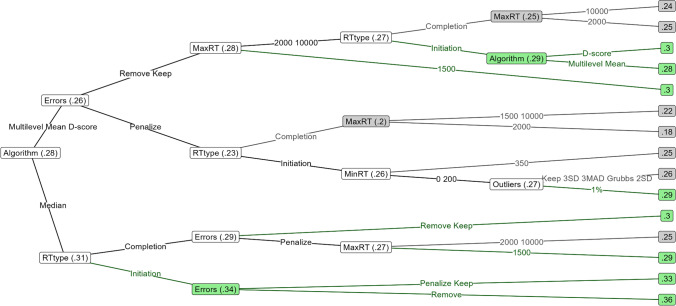
Decision tree of factors influencing the criterion validity of the relevant-feature AAT, as computed with category-specific difference scores

**Fig. 16:**
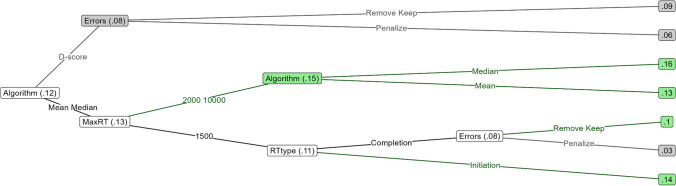
Decision tree of factors influencing the criterion validity of the irrelevant-feature AAT, as computed with category-specific difference scores

## Data Availability

The datasets generated and/or analyzed in the current study can be found in this study’s online repository: 10.17605/OSF.IO/YFX2C

## Appendix 1

### Parameter retrieval procedure

Parameters were computed for the six datasets with and without errors and outliers (defined as RTs below 200 ms or above 2000 ms). For the main effect of movement direction, we computed per participant the mean difference for approach minus avoid trials; for the main effect of stimulus category, we computed the mean difference for trials featuring the target minus control stimuli. For the effect of bias score, we computed the mean difference between trials featuring approach of target stimuli and avoidance of control stimuli, minus trials featuring avoidance of target stimuli and approach of control stimuli. For RT mean and SD, we computed the mean and SD of each participant’s RTs before and after the subtraction of the aforementioned movement, stimulus, and bias effects from the RTs. After this, parameter means and SDs were computed across participants. These parameters are reproduced in Appendix Tables 9 and 10.

**Table 9:** Sample characteristics of the six datasets used in the study

Content	Tasktype	Outliers	N subjects	Mean N trials	Mean errors	Mean RT	Mean RT var.	Full RT SD	Full RT SD var.	Residual RT SD	Residual RT SD var.
Erotica	Relevant-feature	Raw	58	160	10.5	538.09	77.1	175.79	80.81	171.35	79.46
		Clipped	58	148.91	-	536.15	68.98	150.67	44.41	146.31	43.9
	Irrelevant-feature	Raw	58	160	13.62	617.42	107.52	213.55	108.77	211.19	108.07
		Clipped	58	145.5	-	608.04	95.19	180.41	63.29	177.92	62.97
Foods	Relevant-feature	Raw	36	255.75	24.42	618.46	96.93	203.2	79.46	196.9	78.45
		Clipped	36	231.33	-	632.24	90.07	165.87	50.72	158.37	49.87
	Irrelevant-feature	Raw	44	241.39	34.36	527.26	106.84	187.36	80.14	185.14	79.66
		Clipped	44	207.02	-	535.23	97.34	158.09	55.09	155.42	54.4
Spiders	Relevant-feature	Raw	85	128	2.42	548.22	90.57	147.1	71.31	140.69	67.55
		Clipped	85	121.16	-	561.47	76.93	124.49	43.57	117.5	41.68
	Irrelevant-feature	Raw	86	128	5.31	539.46	72.39	124.33	68.24	119.51	65.75
		Clipped	86	122.15	-	539.16	68.82	114.47	42.85	109.57	41.94

**Table 10:** Effect size means and variances of RT contrasts in the six datasets used in the study

Content	Tasktype	Outliers	Pull effect	Pull effect var.	Pull effect size	Stim. effect	Stim. effect var.	Stim. effect size	Bias effect	Bias effect var.	Bias effect size
Erotica	Relevant-feature	Raw	−20.12	36.56	−.55	−16.92	40.51	−.42	26.12	52.22	.5
		Clipped	−25.87	36.78	−.7	−18.58	35.13	−.53	20.99	37.63	.56
	Irrelevant-feature	Raw	−19	38.21	−.5	15.93	37.75	.42	−.06	33.83	0
		Clipped	−28.57	33.32	−.86	11.36	25.81	.44	3.33	31.79	.1
Foods	Relevant-feature	Raw	−30.62	35.24	−.87	−30.88	36.21	−.85	39.26	69.91	.56
		Clipped	−39.21	40.5	−.97	−30.94	32.5	−.95	38.97	60.13	.65
	Irrelevant-feature	Raw	−27.61	38.91	−.71	−4.52	26.41	−.17	1.01	25.6	.04
		Clipped	−33.08	34.45	−.96	−1.18	29.1	−.04	1.22	23.66	.05
Spiders	Relevant-feature	Raw	−27.04	45.96	−.59	−25.05	45.02	−.56	−7.81	62.48	−.12
		Clipped	−31.99	39.16	−.82	−27.77	34.28	−.81	−7.46	52.67	−.14
	Irrelevant-feature	Raw	−35.84	42.22	−.85	11.52	39.22	.29	−5.5	35.56	−.15
		Clipped	−34.93	36.94	−.95	10.52	35.22	.3	−8.31	26.94	−.31

## Appendix 2

### Additional findings in Study 3

#### Category-specific difference scores and the impact of outliers and outlier removal

Category-specific difference scores showed the same pattern as double-difference scores in how they were impacted by outliers and outlier rejection in how well they were able to recover the true score on average, as depicted in Appendix Fig. 8.

#### Variability in true score recoverability of double-difference scores

We also computed the SD, rather than the mean, of the true score recoverability. The SD was computed on the basis of Fisher r-to-z transformed correlations, rather than untransformed correlations. The transformation was applied to minimize the influence of average correlation magnitude on correlation dispersion. The resulting SD represents how unpredictable the correlation between the computed and true score is. The results are depicted in Appendix Fig. 9. They reveal that multilevel compatibility scores are highly variable in their correlation with the true score, compared to the other methods. The results also highlight that median-based scores are not more stable than mean-based scores; on the contrary, D-scores had the smallest SD of their correlation with the true score.

#### Outlier detection rates of outlier exclusion methods given varying numbers of outliers

The outlier detection rates of different outlier rejection procedures are depicted in Appendix Fig. 10. The percentile method stands out as having the highest false negative rate of all outlier detection methods after the data contain more than 1% of outliers, which makes sense, but also the highest false positive rate with fast outliers, which may be because it detects outliers across the entire sample and not within participants. Rejecting RTs deviating more than 2 SD from the participant mean led to the highest true positive rates and lowest false negative rates for fast RTs (i.e. highest sensitivity). For slow RTs, however, the 2 SD method had the highest false positive rate when there were very few outliers, which was apparently to no detriment to the reliability of the data, and this false positive rate was greatly reduced when there were more outliers in the data.

#### Spurious correlates of bias score algorithms

We not only computed correlations between the computed bias score and true underlying bias score, but also with other parameters that were used to generate the data, including true mean RT, true movement direction effect (irrespective of stimulus), and true stimulus category effect (irrespective of movement direction).

The results at an outlier count of 0 are depicted in Appendix Fig. 11. Three aspects of these results are worth noting. First, target-specific bias scores are unsurprisingly correlated with movement direction effects. Second, only the double mean difference score and the double-difference D-score consistently have a decent correlation with the true bias effect, while all other algorithms had below-zero correlations on some occasions. Third and most importantly, the correlation between multilevel-based scores and mean RT is highly spread out both in the positive and negative directions, with almost perfect correlations within the realm of possibility; this is not a property of the data, given that other bias scores do not feature such extreme correlations. This contamination is sure to reduce the validity of multilevel bias scores as well as make them artificially reliable, given that mean RT is a highly reliable variable.

## Appendix 3

### Determining the ideal number of split-halves

We next determined the ideal number of split-halves to use for real data, as there are, to our knowledge, no recommendations on this. We split the six real datasets 100,000 times, computed bias scores for both halves in each split, and recorded the correlation between scores for both halves. From this large pool of split-half correlations, we added one random correlation at a time to a pool and averaged the correlations in the pool together, recording the resulting aggregated correlation for each pool size from 1 to 20,000. This was done 200 times for double mean difference scores, double median difference scores, and double-difference D-score. To analyze the accuracy associated with each pool size, we computed the absolute difference between the average correlation for the pool and that for the entire set of 100,000 splits. For each pool size we counted the number of average correlations deviating more than .005 from the grand average. We computed for each of the six datasets and three algorithms the largest number of iterations below which more than 5% of split halves deviated more than .005 from the grand average. If less than 5% of averages deviated more than .005 from the grand average, this was deemed an acceptable number of splits.

Appendix Fig. 12 depicts the gradual increase in accuracy in split-half reliability estimation as more split-halves were averaged together. Appendix Table 11 depicts the largest number of iterations above which less than 95% of average sets deviated less than .005 from the grand mean of split-half correlations for each scoring algorithm and dataset. To obtain accurate split-half estimates, D-scores required the least iterations, as did the relevant-feature AATs, which tend to be more reliable—for these, 2000 iterations would be more than enough. Mean and median double-difference scores in irrelevant-feature AAT datasets may require more than 5500 split-half iterations to obtain stable results.

**Table 11:** Largest number of pooled split-half correlations at which more than 5% of pool averages deviated more than .005 from the grand average

dataset	Double mean difference	Double median difference	Double-difference D-score
Irrelevant-feature Erotic	2340	2800	1980
Irrelevant-feature Food	3460	5180	1740
Irrelevant-feature Spider	1580	1500	860
Relevant-feature Erotic	1480	1320	700
Relevant-feature Food	540	720	340
Relevant-feature Spider	380	560	280

## Appendix 4

### Decision trees for category-specific scores

For category-specific difference scores, we generated decision trees in the exact same manner as was described in Study 4. The only difference in methodology was that bias scores were computed with only the target stimuli, thus ignoring the control stimuli. Reliability outcomes for the relevant-feature AAT are depicted in Appendix Fig. 13, and for the irrelevant-feature AAT in Appendix Fig. 14. Criterion validity outcomes for the relevant-feature AAT are depicted in Appendix Fig. 15, and for the irrelevant-feature AAT in Appendix Fig 16.
